# Recent Advances in Carboxymethyl Cellulose-Based Solid Polymer Electrolytes Incorporating Lithium Salts and Functional Additives: A Systematic Review

**DOI:** 10.3390/polym18151925

**Published:** 2026-08-05

**Authors:** Asep Muhamad Samsudin, Ridho Prasetyo, Nur Rokhati, Sun Theo Constan Lotebulo Ndruru, Muhammad Aziz, Viktor Hacker

**Affiliations:** 1 Department of Chemical Engineering, Faculty of Engineering, Universitas Diponegoro, Semarang 50275, Indonesia; asep.samsudin@live.undip.ac.id (A.M.S.);; 2 Membrane Research Center, Integrated Laboratory for Research and Services, Universitas Diponegoro, Semarang 50275, Indonesia; 3 Research Center for Molecular Chemistry, Research Organization for Nanotechnology and Materials, National Research and Innovation Agency (BRIN), B.J. Habibie Science and Technopark, South Tangerang 15413, Indonesia; 4 Graduate School of Environmental Studies, Tohoku University, 6-6-20 Aramaki, Aoba-ku, Sendai 980-8579, Japan; 5 Institute of Chemical Engineering and Environmental Technology, Graz University of Technology, 8010 Graz, Austria

**Keywords:** carboxymethyl cellulose, solid polymer electrolyte, lithium salts, ionic conductivity, ion transport, electrochemical stability, degradation temperature, sustainable batteries

## Abstract

Carboxymethyl cellulose (CMC)-based solid polymer electrolytes (SPEs) have attracted significant attention as sustainable alternatives to conventional liquid electrolytes due to their biodegradability, non-toxicity, and excellent film-forming capability. However, pristine CMC suffers from inherent limitations, including low ionic conductivity, poor mechanical strength, and limited electrochemical stability. This systematic literature review comprehensively evaluates recent advances in CMC-based SPEs, focusing on the roles of lithium salts (e.g., LiCH_3_COO, LiClO_4_, LiI, LiBF_4_, and LiNO_3_) and functional additives, including plasticizers, ionic liquids, nanofillers, and cross-linking agents, in tailoring the physicochemical and electrochemical properties. The findings reveal that ionic conductivity can be significantly enhanced from ~10^−7^ to 10^−2^ S cm^−1^ through synergistic modifications that reduce crystallinity and promote segmental mobility. Electrochemical stability is improved by up to ~3.85 V with the incorporation of ionic liquids, while ion transference numbers approaching unity (t^+^ ≈ 0.96) indicate highly efficient Li^+^-dominated transport. Mechanical properties exhibit a trade-off between flexibility (elongation up to ~699%) and tensile strength (up to ~12.84 MPa), depending on the balance between plasticization and cross-linking. The degradation temperature is also strongly influenced by system composition, reaching ~508 °C in ionic liquid-modified systems. Overall, the performance of CMC-based SPEs is governed by the interplay between salt chemistry, polymer structure, and additive functionality. This review highlights key structure–property relationships and identifies critical research gaps, including salt-concentration optimization, long-term stability, and scalability, providing strategic insights for the rational design of high-performance, sustainable polymer electrolytes for next-generation energy storage applications.

## 1. Introduction

The growing global demand for sustainable, high-performance energy storage systems has accelerated the development of safer, more efficient electrolyte materials. Conventional liquid electrolytes suffer from several inherent limitations, including leakage, flammability, and low thermal stability, which restrict their application in advanced electrochemical devices. Consequently, polymer electrolytes have emerged as promising alternatives due to their superior safety, mechanical flexibility, and thermal stability [1,2,3]. Among various polymer hosts, carboxymethyl cellulose (CMC) has gained considerable attention as a biopolymer electrolyte matrix owing to its biodegradability, abundance, non-toxicity, and excellent film-forming ability, as well as the presence of functional groups that facilitate ion transport [4,5,6].

Carboxymethyl cellulose (CMC) is a water-soluble, bio-based polysaccharide derivative characterized by abundant carboxymethyl (–CH_2_–COO^−^) and hydroxyl (–OH) functional groups along its backbone [7]. These intrinsic properties have positioned CMC as a promising host polymer for advanced functional materials, particularly in electrochemical energy storage [8]. In the context of growing global demand for safer, more sustainable batteries, the development of solid polymer electrolytes (SPEs) based on bio-derived polymers, such as CMC, has attracted increasing research attention [9,10].

**Figure 1:**
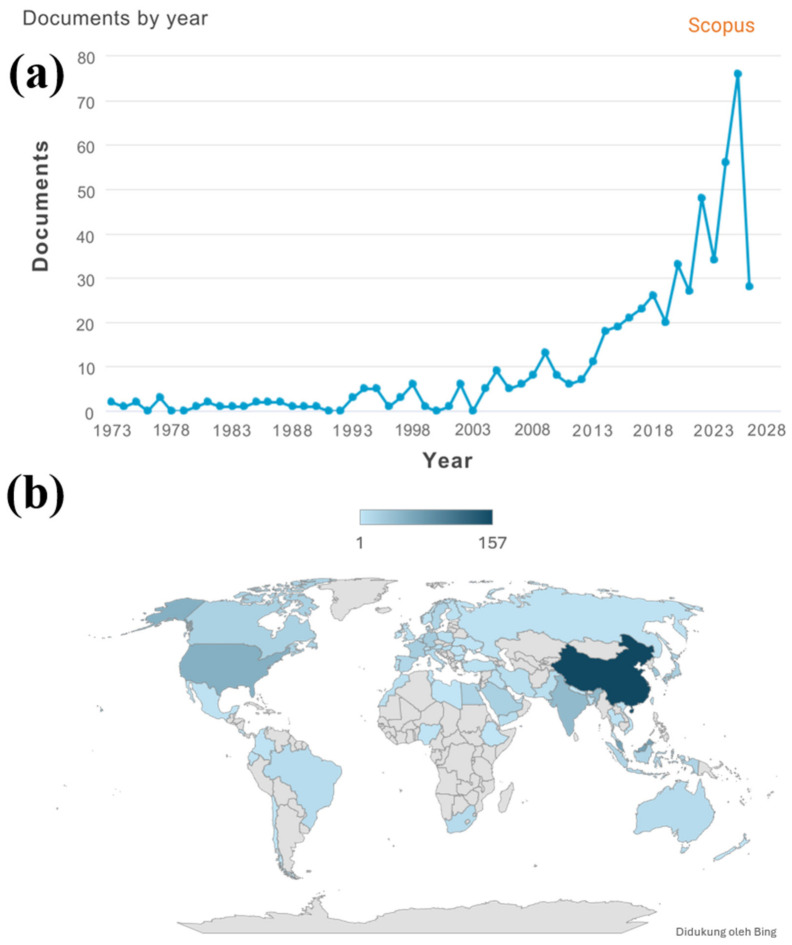
Bibliometric analysis of publications retrieved from Scopus (1973–2025) using the following search query: TITLE-ABS-KEY ((“carboxymethyl cellulose” OR “CMC” OR “carboxymethylcellulose”) AND (“polymer electrolyte” OR “electrolyte polymer” OR “ionic conductor” OR “conductive polymer”)). (**a**) Annual publication trend and (**b**) geographical distribution of publications by country (source: www.scopus.com).

Figure 1 reveals several noteworthy trends beyond the overall increase in publication output. Although research on CMC-based polymer electrolytes began in the 1970s, publication activity remained relatively limited for several decades before increasing rapidly after approximately 2015, with the highest annual output recorded in recent years. This sharp growth reflects the increasing research interest in sustainable polymer electrolytes driven by advances in solid-state batteries and bio-based energy storage materials. Geographically, research activity is dominated by China, followed by Malaysia, the United States, and India, demonstrating broad international interest in this area and highlighting particularly strong contributions from Asia. Despite this rapid expansion, studies remain highly diverse in terms of polymer composition, lithium salt selection, functional additives, and performance evaluation methods. Such diversity makes direct comparisons across studies challenging and underscores the need for a systematic review to critically examine the relationships among material design, physicochemical properties, and electrochemical performance.

Several review articles have summarized the development of CMC-based polymer electrolytes for electrochemical devices. For example, Arif et al. (2025) reviewed the preparation and application of CMC-based polymer electrolyte membranes for energy devices, with emphasis on membrane fabrication strategies and electrochemical applications [10]. Meanwhile, Tyagi et al. (2023) focused on CMC-based materials, covering their broad applications across various fields [11]. Akhlaq et al. (2023) focused on CMC-based energy devices, including applications beyond SPEs [12]. Other reviews have also discussed bio-based polymer electrolytes from a broader perspective [13]. A comparison of the scope, primary focus, and remaining knowledge gaps of these representative review articles is summarized in Table 1. Collectively, these reviews provide valuable insights into sustainable polymer electrolyte development.

Despite these important contributions, several knowledge gaps remain. Most existing reviews primarily provide narrative summaries of material preparation, membrane fabrication, or general applications, whereas a systematic evaluation of how lithium salts and functional additives influence the physicochemical properties, electrochemical performance, and battery applications of CMC-based solid polymer electrolytes is still lacking. In addition, the relationships between electrolyte composition, structure, and performance, as well as the trade-offs among ionic conductivity, mechanical strength, thermal stability, electrochemical stability, and ion transport, have not been comprehensively analyzed. These gaps motivate the present systematic review.

From a materials perspective, CMC-based polymer electrolytes continue to face several critical challenges, including low ionic conductivity at room temperature, limited mechanical strength, and insufficient thermal stability. These limitations hinder their practical application in energy storage devices such as batteries and supercapacitors. Therefore, effective material engineering strategies are required to overcome these constraints. The amphiphilic nature of CMC, combined with its ability to form extensive hydrogen-bonding networks, enables effective blending with various components, including lithium salts [14], plasticizers [15], nanofillers [16], and cross-linking agents [17]. These interactions play a crucial role in modulating the physicochemical properties of the resulting electrolyte system, including crystallinity, segmental mobility, dielectric behavior, and ultimately ionic conductivity. For instance, incorporating lithium salts such as LiClO_4_ [18], LiTFSI [19], or LiPF_6_ [20] provides charge carriers, while additives such as glycerol or polyethylene glycol (PEG) can enhance polymer chain flexibility and reduce the glass transition temperature [21]. Similarly, inorganic nanofillers (e.g., SiO_2_, TiO_2_, and Al_2_O_3_) and cross-linkers (e.g., citric acid) are often incorporated to enhance mechanical strength, thermal stability, and ion-transport pathways [16,22].

To address the knowledge gaps identified above, this systematic review comprehensively examines recent advances in CMC-based solid polymer electrolytes (SPEs), with particular emphasis on the roles of lithium salts and functional additives. Unlike previous reviews that primarily focus on material preparation or general applications, the present review systematically evaluates how lithium salts, plasticizers, ionic liquids, nanofillers, and crosslinking agents influence the physicochemical properties, electrochemical performance, and battery applications of CMC-based SPEs. In addition, this review critically discusses the structure–property–performance relationships and the trade-offs among ionic conductivity, mechanical strength, thermal stability, and electrochemical stability to provide insights into the rational design of next-generation sustainable polymer electrolytes.

It should be noted that CMC-based electrolyte systems reported in the literature encompass several classes of polymer electrolytes with distinct physicochemical characteristics. Dry solid polymer electrolytes (SPEs) contain no intentionally added liquid phase and rely primarily on ion transport through the polymer matrix [23]. In contrast, plasticized polymer electrolytes incorporate low-molecular-weight plasticizers to enhance polymer chain mobility, whereas gel polymer electrolytes (GPEs) contain a substantial amount of liquid electrolyte immobilized within the polymer network [24]. Quasi-solid polymer electrolytes occupy an intermediate state in which limited amounts of plasticizers, ionic liquids, or residual solvents are incorporated while maintaining the mechanical integrity of a solid membrane [25]. Because these electrolyte systems differ in their ion-transport mechanisms, mechanical behavior, and electrochemical performance, the discussions throughout this review explicitly distinguish their characteristics where appropriate.

Compared with previous reviews that focus on biopolymer electrolytes in general, this review specifically emphasizes CMC-based solid polymer electrolytes and provides a comprehensive analysis of how lithium salts and functional additives affect their structure–property relationships, electrochemical performance, and future design strategies.

## 2. Methodology of the Systematic Review

### 2.1. Literature Search Strategy

This systematic literature review was conducted in accordance with the Preferred Reporting Items for Systematic Reviews and Meta-Analyses (PRISMA 2020) guidelines. Scopus was selected as the sole database because of its comprehensive coverage of peer-reviewed publications in materials science, polymer chemistry, electrochemistry, and energy storage.

The literature search was performed in three sequential stages to identify studies on carboxymethyl cellulose (CMC)-based solid polymer electrolytes (SPEs) for lithium-ion batteries. Initially, a broad search was conducted using the following query: TITLE-ABS-KEY ((“carboxymethyl cellulose” OR “CMC” OR “carboxymethylcellulose”) AND (“polymer electrolyte” OR “electrolyte polymer” OR “ionic conductor” OR “conductive polymer”)). This search retrieved 243 records describing the general development of CMC-based polymer electrolytes, as shown in the bibliometric analysis in Figure 1.

**Figure 2:**
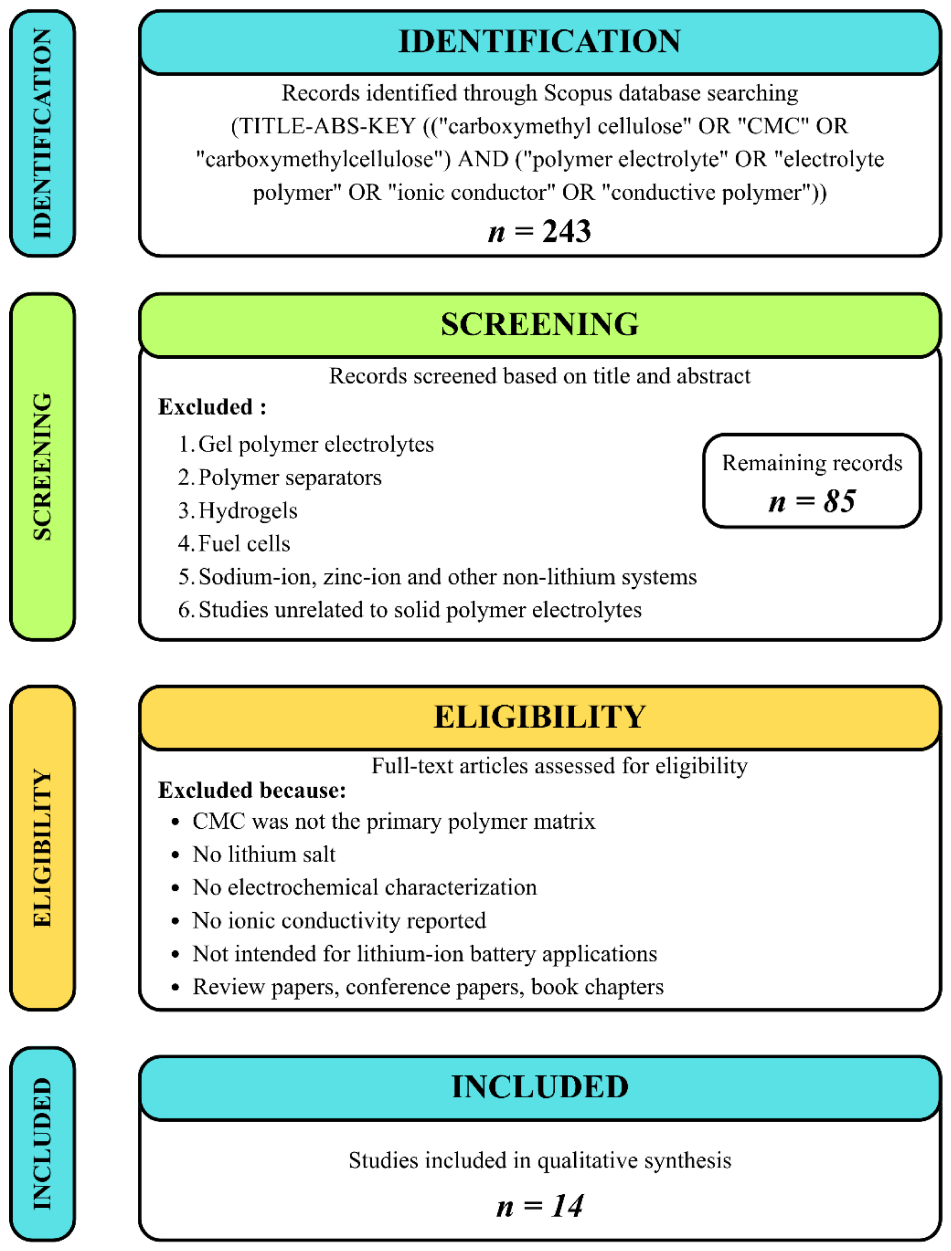
PRISMA 2020 Flow Diagram of The Literature Selection Process.

Subsequently, the records were screened to retain only studies involving solid polymer electrolytes (SPEs) and to exclude gel polymer electrolytes, battery separators, hydrogels, composite membranes unrelated to SPEs, and publications outside the scope of lithium-ion battery electrolytes. After this screening process, 85 articles remained. Finally, a more specific search was performed to identify studies reporting the electrochemical performance of CMC-based SPEs for lithium-ion batteries using the following query: TITLE-ABS-KEY ((“solid polymer electrolyte*” OR “polymer electrolyte*” OR “SPE”) AND (“carboxymethyl cellulose” OR “CMC”) AND (“lithium salt*” OR “lithium ion” OR “Li+”) AND (“properties” OR “performance” OR “characteristics” OR “conductivity” OR “electrochemical stability” OR “ionic conductivity”) AND (“lithium-ion battery*” OR “Li-ion battery*” OR “lithium battery*” OR “energy storage”)). This search identified 14 eligible studies, which formed the final dataset for qualitative synthesis and comparative analysis. The complete identification, screening, eligibility assessment, and inclusion process is presented in the PRISMA 2020 flow diagram (Figure 2).

### 2.2. Eligibility Criteria

The inclusion and exclusion criteria were established prior to the screening process to ensure consistency during article selection, as shown in Table 2.

### 2.3. Study Selection

The study selection process followed the PRISMA 2020 workflow. All records obtained from the initial database search were screened based on titles and abstracts. Duplicate records, irrelevant publications, and studies that did not meet the inclusion criteria were excluded during the screening stage. The remaining articles underwent full-text assessment to determine their eligibility.

The selection process consisted of three sequential phases:

1. Identification of 243 publications from the Scopus database.
2. Topic refinement to studies focusing specifically on solid polymer electrolytes, resulting in 85 publications.
3. Full-text eligibility assessment based on predefined inclusion criteria, yielding 14 studies for qualitative synthesis.

A PRISMA flow diagram illustrating the complete screening procedure is presented in Figure 2.

### 2.4. Data Extraction

Relevant information from the selected studies was systematically extracted using a standardized data extraction form. The extracted variables included publication year, polymer composition, lithium salt, plasticizer, inorganic filler, preparation method, ionic conductivity, electrochemical stability window, thermal properties, crystallinity, mechanical properties, battery performance, and principal findings.

These data were synthesized to identify current research trends, compare electrolyte performance, evaluate relationships between material composition and electrochemical behavior, and determine existing research gaps for future development of CMC-based solid polymer electrolytes.

### 2.5. Data Synthesis

A qualitative synthesis approach was employed to compare the characteristics and performance of the selected studies. Comparative analysis focused on the influence of polymer composition, lithium salt type, plasticizer content, and filler incorporation on ionic conductivity, structural properties, and electrochemical performance.

The synthesized evidence was subsequently organized into thematic categories, including polymer matrix design, ionic transport mechanisms, physicochemical properties, electrochemical performance, and future research opportunities. This approach enabled the identification of technological trends, current limitations, and promising strategies for developing sustainable CMC-based solid polymer electrolytes for lithium-ion battery applications.

## 3. Fundamentals of CMC-Based Polymer Electrolytes

### 3.1. Structure and Properties of CMC

**Figure 3:**
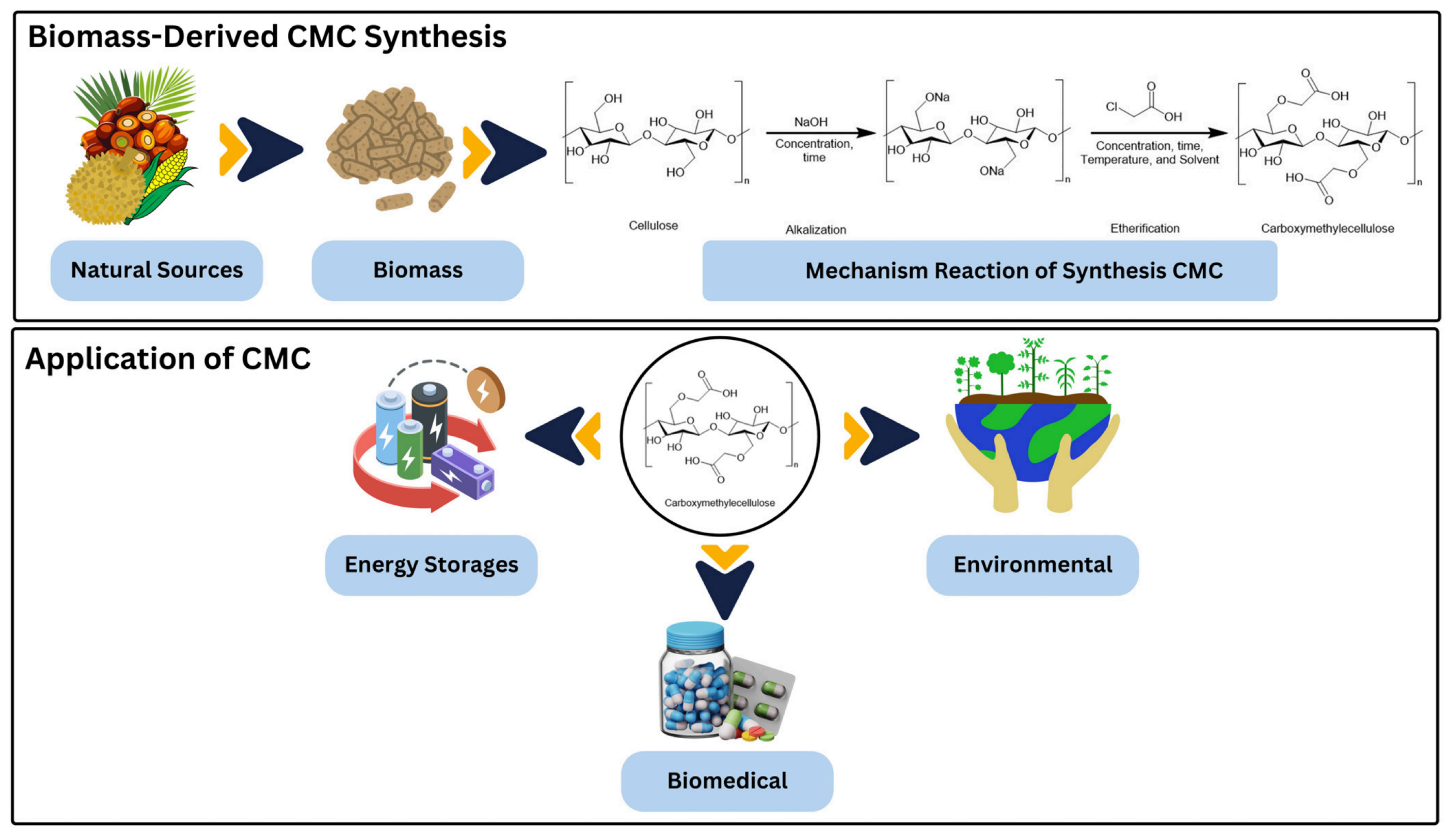
Schematic illustration of the conversion of biomass into carboxymethyl cellulose (CMC) and its representative applications.

Carboxymethyl cellulose (CMC) is a cellulose derivative that has attracted considerable attention due to its diverse physicochemical properties and wide range of applications. Structurally, CMC is produced through the carboxymethylation of cellulose, in which carboxymethyl groups (CH_2_–COOH) are introduced into the cellulose backbone, particularly in the amorphous regions, thereby enhancing its solubility and reactivity compared to native cellulose [7,26]. The presence of these functional groups imparts an anionic character to CMC, making it amphiphilic and responsive to environmental conditions such as pH and ionic strength [27]. In addition, CMC can be synthesized from various biomass wastes, including durian peel [28], oil palm empty fruit bunches [29], and other lignocellulosic materials (Figure 3). An important structural parameter is the degree of substitution (DS), which represents the average number of carboxymethyl groups per anhydroglucose unit. Higher DS values generally lead to improved solubility, viscosity, and film-forming ability [30]. Furthermore, the supramolecular structure of CMC is influenced by pH, where electrostatic repulsion between charged groups can cause polymer chain expansion at specific pKa conditions [31].

In terms of properties, CMC exhibits high water solubility due to the presence of hydrophilic functional groups and shows pseudoplastic behavior, in which viscosity decreases under shear stress, depending on concentration and temperature [11,32]. It also possesses excellent film-forming properties, producing transparent, biodegradable films with stable internal structures, making it suitable for packaging applications [32]. From a mechanical standpoint, CMC-based materials exhibit adequate tensile strength and elongation, which can be further enhanced by blending with other polymers or by cross-linking to improve flexibility and thermal stability. Chemically, CMC is biodegradable, biocompatible, and non-toxic, making it suitable for biomedical, environmental, and energy storage applications.

### 3.2. Limitations of Pure CMC Electrolyte

Carboxymethyl cellulose (CMC) is widely used in solid polymer electrolytes (SPEs) due to its renewability, hydrophilicity, and excellent film-forming properties. However, pure CMC exhibits several inherent limitations that restrict its performance in energy storage applications. One of the main challenges is its relatively low ionic conductivity, typically in the range of 10^−6^ S/cm, which is insufficient for practical use [18,33]. This limitation is closely related to its high crystallinity, which reduces the formation of amorphous regions necessary for efficient ion transport [10,34]. In addition, pure CMC demonstrates poor mechanical and thermal stability, with degradation occurring at relatively low temperatures and insufficient tensile strength for robust electrolyte membranes [35,36]. Its highly hydrophilic nature also makes it sensitive to environmental conditions, particularly moisture, which can affect electrochemical stability and overall performance [37]. Furthermore, the electrochemical stability window of pure CMC is generally limited to below 3 V, restricting its applicability in high-voltage systems [38]. Compatibility issues, such as dissolution in aqueous environments and the presence of impurities, further contribute to performance degradation [39].

To overcome these limitations, various modification strategies have been extensively explored. Polymer blending, such as combining CMC with polyvinyl alcohol (PVA) or chitosan, has been shown to enhance mechanical properties and ionic conductivity [40,41,42]. The incorporation of salts or ionic liquids, including LiTFSI and NH_4_SCN, can significantly improve ionic conductivity and broaden electrochemical stability [43,44]. Cross-linking using agents such as citric acid or polycarboxylic acids is another effective approach to increase thermal stability and mechanical strength [17,45]. Additionally, reducing CMC crystallinity through chemical or physical treatments can increase the proportion of amorphous regions, thereby facilitating ion transport and improving overall electrolyte performance [34]. These strategies highlight the importance of material engineering in optimizing CMC-based SPEs for advanced energy storage applications.

## 4. Recent Synthesis Methods and Material Design

### 4.1. Solution Casting

The solution-casting method is the most widely used technique for preparing carboxymethyl cellulose (CMC)-based solid polymer electrolytes (SPEs). This method involves dissolving CMC along with lithium salts and various additives in water or other suitable solvents to form a homogeneous solution [46]. The resulting solution is then cast onto a flat surface or mold and subjected to controlled drying to produce a solid electrolyte film. The main advantages of this approach include its procedural simplicity, low cost, and ease of controlling the composition of the constituent materials [47]. Furthermore, solution casting enables a relatively uniform distribution of components at the laboratory scale, making it a preferred method in many studies on biopolymer-based SPE development [9,13,48].

Despite these advantages, the solution casting method also has several limitations, particularly regarding microstructural uniformity and scalability. Non-uniform solvent evaporation during drying can lead to the formation of pores, cracks, or phase separation within the electrolyte film, potentially affecting its mechanical properties and ionic conductivity [49,50]. In addition, scaling up the process introduces challenges in maintaining consistent film thickness and reproducible material quality. Therefore, while this method is well-suited for preliminary studies and compositional optimization, further development is needed to improve material consistency and performance, especially for large-scale or industrial applications.

### 4.2. Composite Formation

Composites aim to address the limitations of SPEs, such as low ionic conductivity, poor mechanical strength, and limited electrochemical stability, while leveraging the flexibility and lightweight nature of polymers [51,52]. Composite formations are produced by combining a polymer matrix with other polymers or nanofillers via blending. Blending CMC with other polymers, such as poly(ethylene oxide) (PEO), polyvinyl alcohol (PVA), and methylcellulose, has been widely explored as an effective strategy to enhance the performance of solid polymer electrolytes (SPEs). This approach aims to combine the advantageous properties of each polymer, with CMC providing biodegradability and abundant functional groups, while polymers like PEO provide excellent ion-transport pathways due to their segmental mobility and ether-oxygen coordination sites [53]. Similarly, PVA offers good film-forming ability and mechanical strength, whereas methylcellulose can improve structural stability and compatibility within the polymer matrix [54,55]. Through such polymer blending, the resulting electrolyte system can exhibit improved flexibility, reduced crystallinity, and enhanced ionic conductivity due to increased amorphous regions that facilitate ion migration.

In addition to polymer blending, incorporating nanofillers, such as hectorite and cellulose nanocrystals (CNCs), has been shown to significantly improve the mechanical and electrochemical properties of CMC-based SPEs [46,56]. These nanofillers can act as reinforcing agents, enhancing tensile strength and thermal stability while also contributing to the formation of more efficient ion-transport pathways [57]. The presence of nanofillers can disrupt polymer chain packing, thereby increasing amorphous content and promoting ion mobility [58,59]. Furthermore, specific interactions between the filler surface and the polymer matrix or ionic species can facilitate salt dissociation and reduce ion pairing, thereby increasing ionic conductivity. Therefore, the synergistic combination of polymer blending and nanofiller incorporation represents a promising approach for designing high-performance, flexible, and sustainable polymer electrolytes for advanced energy storage applications.

### 4.3. Cross-Linking

Cross-linking is an important modification strategy for developing CMC-based solid polymer electrolytes (SPEs) to enhance structural integrity and functional performance. This approach involves the use of cross-linking agents such as citric acid [18], 4-(4,6-dimethoxy-1,3,5-triazin-2-yl)-4-methylmorpholinium chloride (DMTMM) [60], ethylene glycol diglycidyl ether (EGDE), vanillin, and multivalent ions such as Fe^3+^, to form either covalent bonds or coordination interactions within the polymer network [61]. Through these interactions, a three-dimensional network is formed, significantly improving the mechanical strength and dimensional stability of the resulting electrolyte membrane. Additionally, cross-linking can reduce excessive swelling and enhance water resistance, which is particularly important for maintaining performance under humid or aqueous conditions [62].

From an electrochemical perspective, cross-linking also plays a crucial role in balancing mechanical robustness and ionic conductivity. While excessive cross-linking may restrict polymer chain mobility and hinder ion transport, an optimal cross-linking density can create a stable yet flexible matrix that supports efficient ion migration [63,64]. Moreover, certain cross-linking agents can introduce additional functional groups or coordination sites that facilitate interactions with lithium ions, thereby promoting salt dissociation and improving ionic conductivity [65,66]. Consequently, the careful selection and optimization of cross-linking agents and conditions are essential to achieving high-performance CMC-based SPEs with improved mechanical, thermal, and electrochemical properties [65,67].

## 5. Classification of Additives in CMC-Based Polymer Electrolytes

### 5.1. Types and Roles of Lithium Salts in CMC-Based SPEs

**Figure 4:**
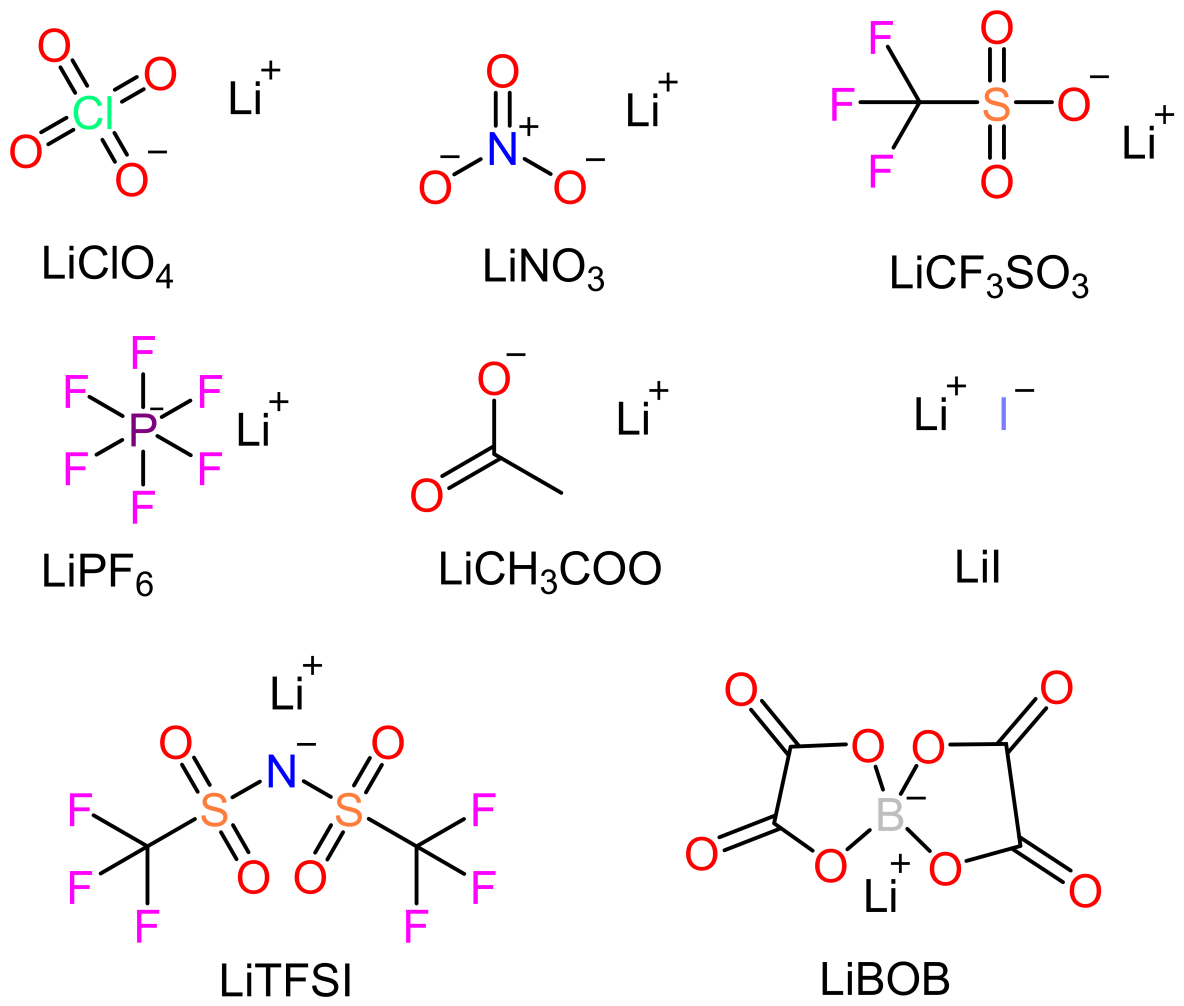
Selected lithium salts to increase the ion conductivity in carboxymethyl cellulose-based solid polymer electrolytes.

The selection of lithium salt plays a critical role in determining the electrochemical performance, ionic conductivity, and stability of CMC-based solid polymer electrolytes (SPEs). Different lithium salts exhibit distinct physicochemical characteristics, such as lattice energy, ion-dissociation ability, and interactions with the polymer matrix, which directly influence ion transport behavior and overall electrolyte performance. Commonly used lithium salts include LiClO_4_ [68], LiNO_3_ [69], LiCF_3_SO_3_, LiCH_3_COO (LiOAc), LiI [70], LiTFSI [71], LiPF_6_ [20], and LiBOB [72], each exhibiting different characteristics in terms of dissociation ability, anion size, and electrochemical stability, as shown in Figure 4.

In general, lithium salts with large, delocalized anions, such as LiTFSI and LiCF_3_SO_3_, tend to dissociate more readily, thereby increasing the number of free ions and enhancing ionic conductivity [73]. In contrast, salts such as LiPF_6_ and LiBOB are often favored for their superior electrochemical stability. In addition to salt type, concentration is also critical, with an optimal range typically 25–30 wt% to balance ionic conductivity and mechanical strength [71]. At the molecular level, Li^+^ ions interact with the carboxylate and ether functional groups of CMC, thereby reducing crystallinity and increasing amorphous regions, which facilitate ion transport [74].

However, excessive salt loading (e.g., LiTFSI > 30 wt%) can result in ion aggregation and salt crystallite formation, which, in turn, reduces both ionic conductivity and mechanical performance [71]. To address this issue, incorporating nanofillers such as ZnO has been shown to disrupt polymer chain ordering, enhance ion migration pathways, and improve mechanical and thermal stability [16]. Nevertheless, increasing salt concentration often introduces a trade-off in the form of reduced mechanical strength, which can be mitigated by adding plasticizers or reinforcing fillers to maintain flexibility and structural integrity [75]. Despite extensive studies, certain systems such as LiBOB in CMC-based matrices remain underexplored, highlighting opportunities for further investigation into optimal composition and structure–property relationships in these electrolyte systems.

### 5.2. Plasticizer

**Figure 5:**
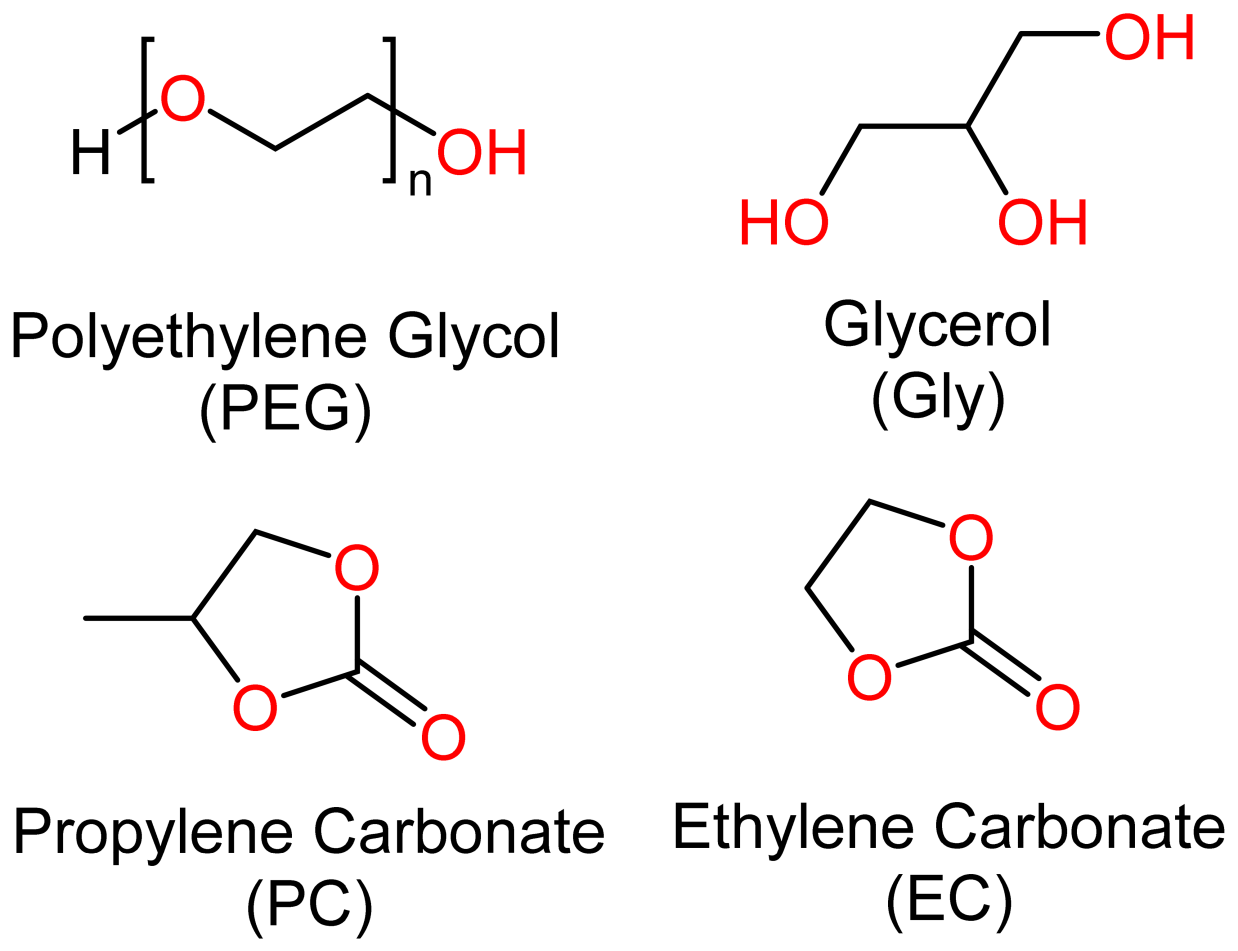
Selected plasticizers to maintain the flexibility and structural integrity of CMC-based SPEs.

Plasticizers are additives used in polymer electrolytes to enhance their flexibility, ionic conductivity, and overall performance in batteries. They help to lower the glass transition temperature of the polymer matrix, making it more pliable and improving ion transport [76]. Common types of plasticizers used in polymer electrolytes for batteries are shown in Figure 5.

Plasticizers play a crucial role in improving the performance of CMC-based solid polymer electrolytes (SPEs) by enhancing polymer chain mobility, reducing crystallinity, and facilitating ion transport. Commonly used plasticizers exhibit distinct physicochemical properties that influence both ionic conductivity and mechanical behavior. Propylene carbonate (PC), for instance, is widely recognized for its high dielectric constant and strong solvating ability for lithium salts, which promote salt dissociation and significantly enhance ionic conductivity [77]. Similarly, ethylene carbonate (EC) not only improves ionic conductivity but also enhances mechanical properties and is often used in combination with other plasticizers to optimize electrolyte performance [78].

Polyethylene glycol (PEG) is another frequently used plasticizer that enhances flexibility and ionic conductivity by acting as a cosolvent for lithium salts and increasing segmental motion within the polymer matrix [79]. Glycerol, a biodegradable and environmentally friendly plasticizer, is particularly attractive for green electrolyte systems, as it effectively increases flexibility and ionic conductivity while maintaining sustainability aspects [80]. Both PEG and glycerol increase the free volume of the polymer matrix and weaken intermolecular interactions, thereby promoting polymer chain mobility and facilitating ion transport.

It should be noted that CMC-based electrolyte systems containing plasticizers are fundamentally different from conventional dry, solvent-free solid polymer electrolytes. The incorporation of plasticizers, such as carbonate solvents, glycerol, or polyethylene glycol, increases polymer chain mobility and free volume, thereby facilitating lithium-ion transport through enhanced segmental motion [81]. Consequently, these systems are more appropriately classified as plasticized or quasi-solid polymer electrolytes rather than completely dry SPEs. Although plasticization generally improves ionic conductivity, excessive plasticizer content may reduce mechanical strength, dimensional stability, and long-term electrochemical durability [54]. Therefore, optimizing both the type and concentration of plasticizers is essential to achieve a balanced combination of high ionic conductivity, mechanical robustness, and operational stability.

### 5.3. Nanofillers

Nanofillers are materials added to polymer electrolytes to enhance their mechanical properties, ionic conductivity, and thermal stability. Particles with a size of 1–100 nm are used in polymer electrolytes, significantly enhancing ionic conductivity by creating free-ion pathways [82,83]. They can significantly improve the performance of polymer electrolytes in lithium-ion batteries.

Nanofillers play a crucial role in the development of CMC-based solid polymer electrolytes (SPEs) by enhancing mechanical properties, thermal stability, and ionic conductivity through modification of the material’s microstructure. The incorporation of nanofillers can disrupt the ordered arrangement of polymer chains, thereby increasing the amorphous fraction and facilitating ion mobility [83]. Silica (SiO_2_), for example, is widely used due to its high surface area, which contributes to improved mechanical strength, enhanced thermal stability, and the formation of additional ion-transport pathways [84]. Similarly, alumina (Al_2_O_3_) improves mechanical properties and thermal stability while helping suppress dendrite growth, which can degrade battery performance [85].

Titanium dioxide (TiO_2_) is another commonly used nanofiller with a high dielectric constant, which promotes salt dissociation, enhances ionic conductivity, and improves electrochemical stability [86]. Zirconia (ZrO_2_) has been shown to increase ionic conductivity and thermal stability, particularly in systems operating at elevated temperatures [59]. Meanwhile, graphene oxide (GO), with its large surface area and abundant oxygen-containing functional groups, can enhance interactions with the polymer matrix, thereby improving the ionic conductivity and mechanical strength of the electrolyte membrane [87].

### 5.4. Ionic Liquid

**Figure 6:**
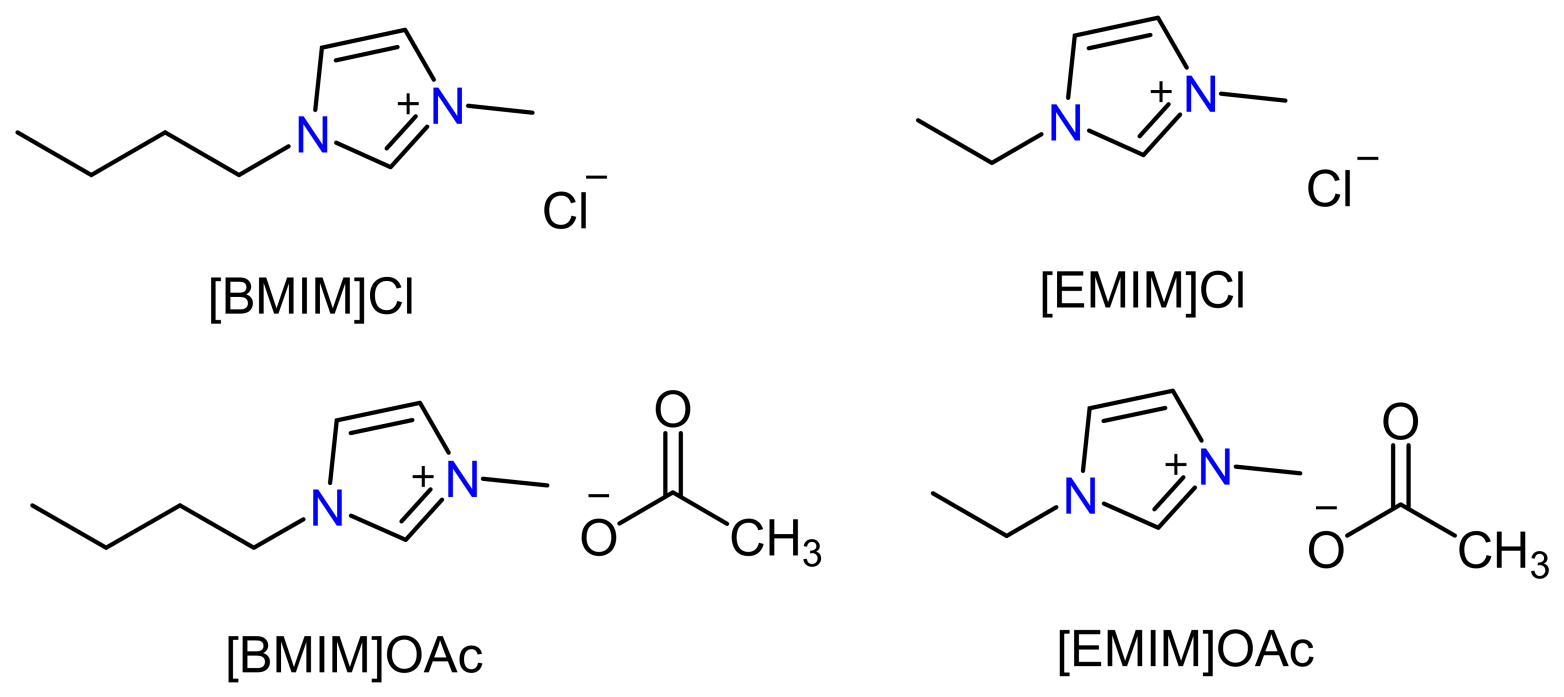
Ionic liquid selected to enhance ionic conductivity of CMC-based solid SPEs.

Ionic liquids (ILs) are salts that are liquid at room temperature and are composed entirely of ions. They have unique properties such as low volatility, high thermal stability, and excellent ionic conductivity, making them suitable for use in polymer electrolytes for batteries [43,88]. Figure 6 shows the common types of ionic liquids utilized in polymer electrolytes.

Ionic liquids are important components in the development of solid polymer electrolytes (SPEs) as they can enhance ionic conductivity, electrochemical stability, and compatibility with polymer matrices. Commonly used ionic liquids include imidazolium-based cations such as BMIM (1-butyl-3-methylimidazolium) and EMIM (1-ethyl-3-methylimidazolium). The BMIM ionic liquid is known for its high ionic conductivity and good compatibility with various polymer matrices, thereby improving the overall electrochemical performance of the electrolyte system [89]. Meanwhile, EMIM also exhibits high ionic conductivity and strong solvation ability for salts and is thus widely used in SPE systems [90].

Incorporating ionic liquids into CMC-based SPE systems offers additional advantages, including greater flexibility, reduced crystallinity, and enhanced thermal and electrochemical stability [91]. Ionic liquids can function as both plasticizers and ion-conducting media, increasing the number of free ions and facilitating ion mobility within the polymer matrix [92]. Furthermore, interactions between ionic liquids and the functional groups of CMC help stabilize the amorphous structure, thereby promoting more efficient ion transport. Therefore, the appropriate selection of ionic liquids is a key strategy for optimizing the performance of CMC-based SPEs, particularly for energy storage applications that require high conductivity and long-term stability.

Similar to plasticized systems, CMC-based electrolytes incorporating ionic liquids should be distinguished from conventional dry SPEs. Ionic liquids not only enhance ionic conductivity by improving lithium salt dissociation and ion mobility, but also contribute to the formation of quasi-solid polymer electrolytes due to the presence of a non-volatile liquid phase within the polymer matrix [43]. Consequently, their ion transport behavior, mechanical properties, and electrochemical performance differ from those of solvent-free SPEs and should be interpreted within the context of quasi-solid electrolyte systems.

### 5.5. Cross-Linker

**Figure 7:**
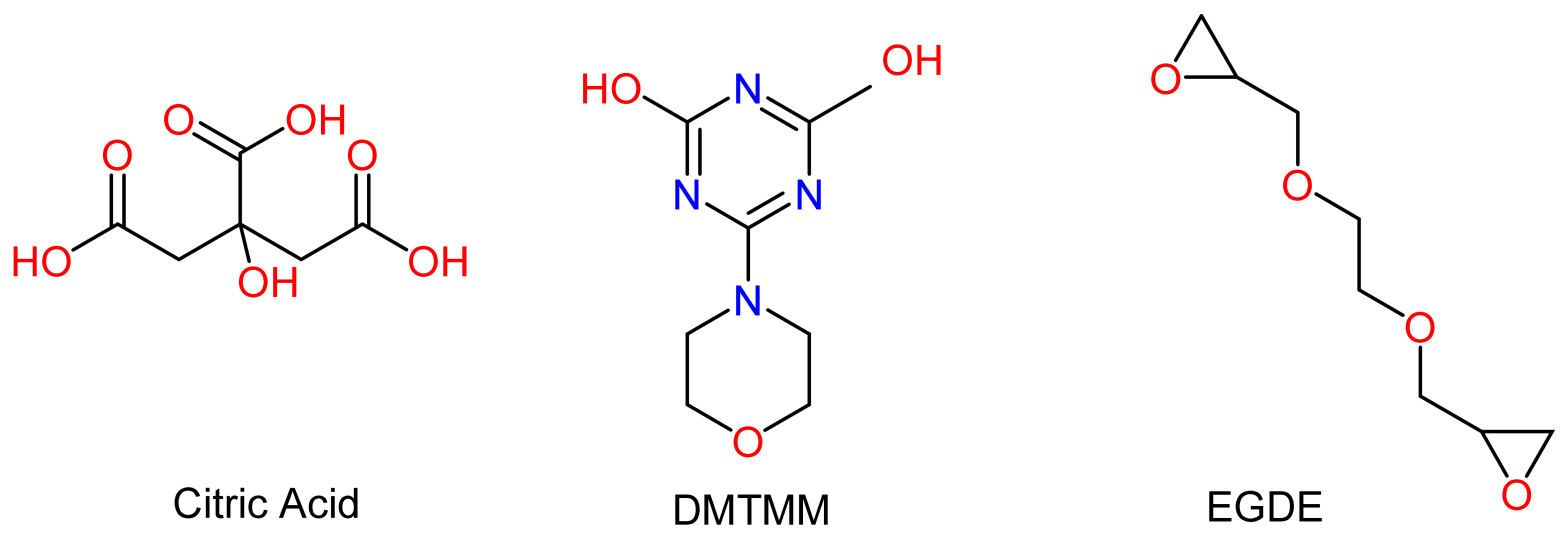
Selected cross-linker to increase the mechanical properties of CMC-based SPEs.

Polymer electrolytes, particularly GPEs and SPEs, rely on cross-linkers to enhance their mechanical, thermal, and electrochemical properties (Figure 7). Cross-linkers are agents that form bonds between polymer chains, thereby creating three-dimensional networks [93]. These bonds can be covalent or ionic, and cross-linking significantly alters the physical, mechanical, and chemical properties of polymers [94,95]. Cross-linkers can be categorized as covalent or non-covalent, with the former being more commonly used in battery applications [96,97].

Cross-linking in CMC-based systems can be achieved through various mechanisms, including both covalent and non-covalent interactions, each contributing differently to the structure and performance of solid polymer electrolytes (SPEs). One of the most commonly used approaches is covalent cross-linking using polycarboxylic acids such as citric acid. This compound contains multiple –COOH groups that can react with –OH groups on CMC through esterification reactions, forming a three-dimensional network (covalent cross-linked network) [17,96]. This structure enhances mechanical strength, improves thermal stability, and reduces the polymer’s water solubility.

In addition, covalent cross-linking can be achieved using highly reactive compounds, such as epoxide-containing agents (e.g., EGDE) or carboxyl-activating agents (e.g., DMTMM) [60]. These reactive groups enable covalent bonding between polymer chains via reactions with hydroxyl or carboxyl groups in CMC. As a result, a more stable, homogeneous network structure forms, which is essential for maintaining the integrity of the electrolyte membrane during operation. On the other hand, cross-linking can also occur through non-covalent interactions, such as ionic or coordination bonding. This approach typically involves multivalent ions such as Fe^3+^ or Ca^2+^, which interact with carboxylate (–COO^−^) groups in CMC to form reversible coordination bonds [61]. Additionally, hydrogen bonding between polymer chains may also contribute to network formation. Although these interactions are weaker than covalent bonds, they provide greater flexibility while still enhancing structural stability and electrolyte performance.

## 6. Effect of Additives on Physicochemical Properties of SPEs

### 6.1. Ion Transport Mechanism

**Figure 8:**
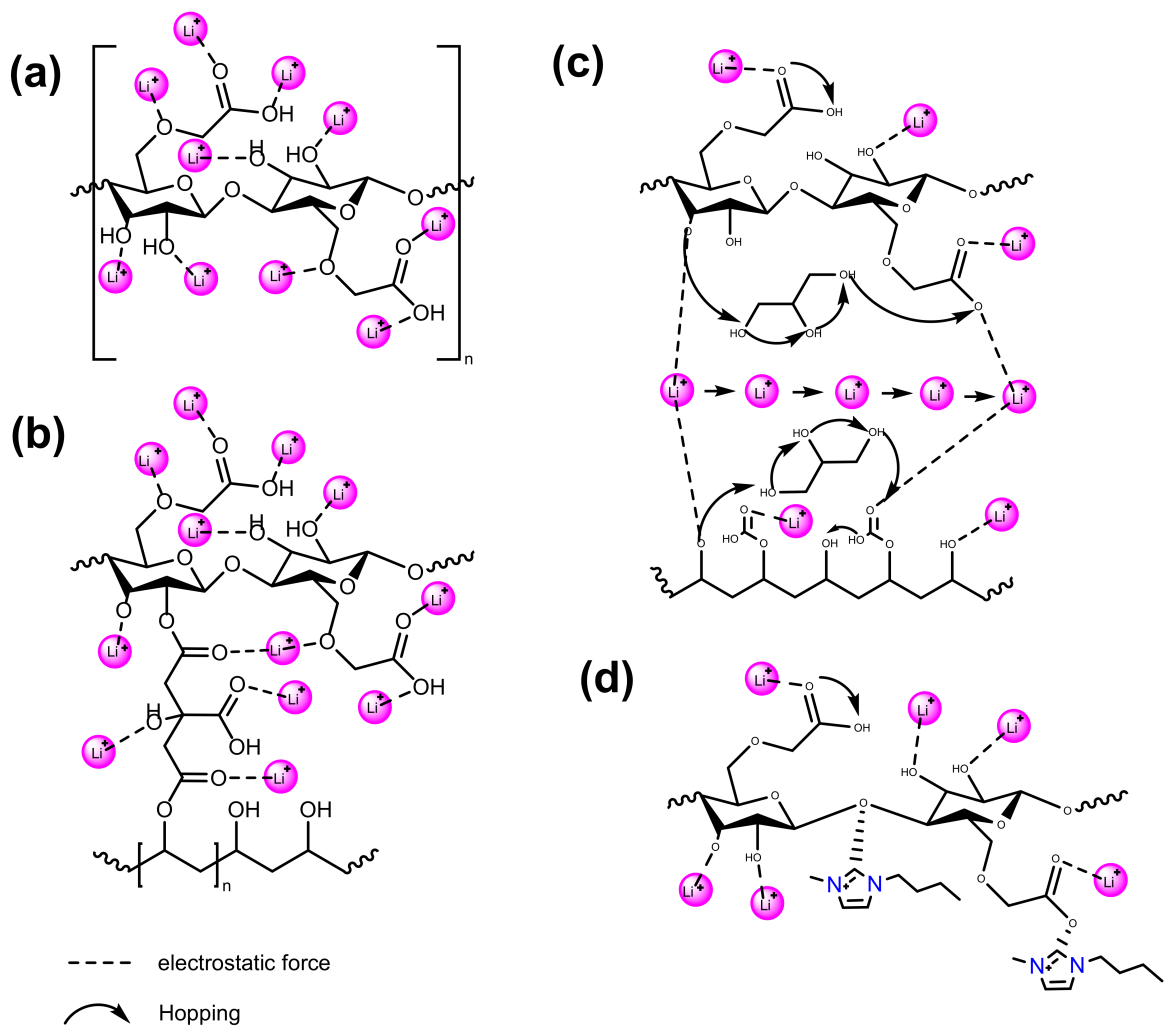
Ion transport mechanism of (**a**) CMC with lithium salt, (**b**) CMC/PVA/Li, (**c**) CMC/PVA/Gly/Li, and (**d**) CMC/[BMIM]/Li.

Carboxymethyl cellulose (CMC) acts as a host polymer in polymer electrolyte systems, where functional groups such as –OH and –COO^−^ along the polymer backbone serve as coordination sites for metal ions, particularly Li^+^, as shown in Figure 8.

Figure 8a illustrates the ion transport mechanism in carboxymethyl cellulose (CMC)-based polymer electrolytes doped with lithium salts, highlighting the dominant role of CMC functional groups in facilitating ionic conduction. In the CMC/LiCH_3_COO system, Li^+^ ions coordinate with oxygen atoms from hydroxyl (–OH) and carboxylate (–COO^−^) groups along the polymer backbone, forming transient coordination bonds that enable a “hopping” mechanism between adjacent coordination sites [98]. The mobility of Li^+^ ions is strongly governed by the segmental motion of the polymer chains, indicating that the flexibility of the CMC matrix is a key factor in enhancing ionic conductivity. In contrast, in the CMC/LiI system, ionic interactions arise from the dissociation of the salt into Li^+^ and I^−^ ions, where Li^+^ remains coordinated to oxygen-containing functional groups of CMC, while the I^−^ anion contributes to charge stabilization through relatively weak electrostatic interactions [70]. The presence of a large anion such as I^−^ can also suppress ion pairing, thereby increasing the number of free charge carriers available for conduction [99].

Meanwhile, in the CMC/PVA/CA/LiClO_4_ system (Figure 8b), citric acid (CA) acts as a cross-linking agent, forming a three-dimensional polymer network between CMC and polyvinyl alcohol (PVA). This cross-linked structure provides interconnected pathways for Li^+^ transport, as illustrated by the hopping trajectory, while maintaining sufficient segmental mobility for ion conduction [17]. Collectively, these systems demonstrate that the combination of polymer blending, functional group density, and cross-linked network formation significantly influences ion transport behavior by modulating coordination sites, reducing crystallinity, and enhancing the continuity of ionic pathways, ultimately leading to improved electrochemical performance.

In the CMC/PVA/Gly/LiClO_4_ system (Figure 8c), glycerol acts as a plasticizer that increases the free volume and reduces intermolecular hydrogen bonding within the polymer matrix, thereby enhancing chain flexibility and facilitating Li^+^ ion coordination with oxygen atoms from both CMC and PVA [80]. This results in a more efficient segmental motion-assisted hopping mechanism. In the CMC/[BMIm]Cl/LiCH_3_COO system (Figure 8d), the ionic liquid 1-butyl-3-methylimidazolium chloride ([BMIm]Cl) promotes salt dissociation. It provides additional mobile ions while disrupting polymer crystallinity, leading to the formation of highly conductive pathways that are generally attributed to the combined effects of vehicular and hopping transport mechanisms [100].

The ion transport mechanisms discussed above are based on the interpretations reported in the literature for representative CMC-based polymer electrolyte systems. It should be recognized that the measured ionic conductivity generally represents the total ionic conductivity of the electrolyte and does not necessarily correspond exclusively to Li^+^ transport. Depending on the electrolyte composition, particularly in systems containing plasticizers, ionic liquids, or high concentrations of lithium salts, both cationic and anionic species may contribute to the overall ionic conduction. Therefore, the proposed hopping and vehicular mechanisms should be regarded as plausible interpretations rather than definitive transport pathways. Direct identification of the dominant ion-transport mechanism requires complementary experimental techniques, such as lithium-ion transference-number measurements, spectroscopic characterization, or molecular simulations.

### 6.2. Crystallinity Index (CrI)

**Figure 9:**
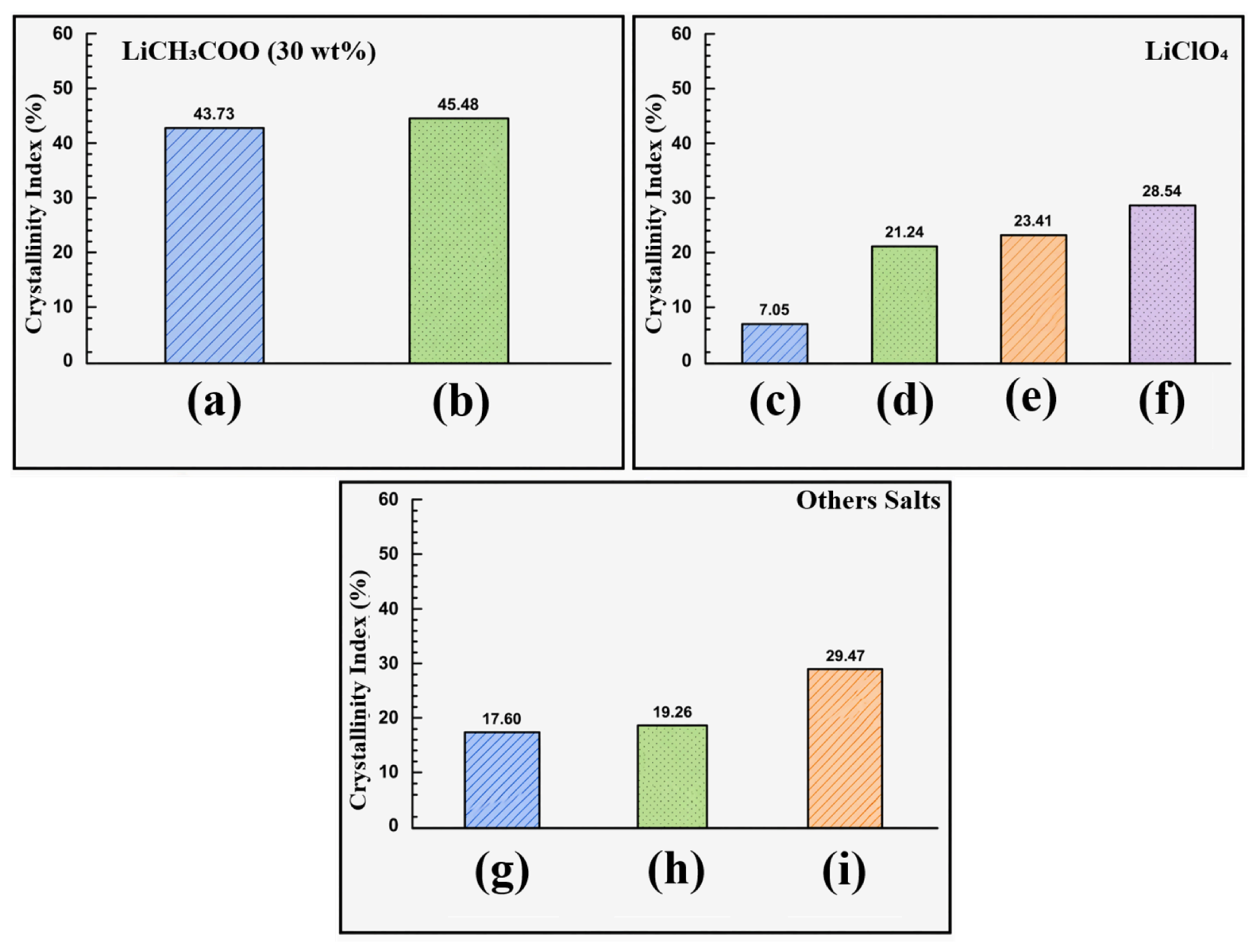
Effect of lithium salts and an additive on crystallinity index (Cr.I): (a) CMC/[BMIm]Cl/LiCH_3_COO [100], (b) CMC/LiCH_3_COO [98], (c) CMC/pectin/Gly/LiClO_4_ [101], (d) CMC/MC/LiClO_4_ [46], (e) CMC/PVA/Gly/LiClO_4_ [80], (f) CMC/PVA/CA/LiClO_4_ [17], (g) CMC/Gly/LiI [102], (h) CMC/LiI [70], and (i) CMC/PVA/LiNO_3_ [103].

The significant influence of lithium salt type and additive addition on the crystallinity index (CrI) of a CMC-based solid polymer electrolyte is shown in Figure 9, and CrI is directly correlated with ion transport properties.

Figure 9 demonstrates the significant influence of lithium salt type and additive incorporation on the crystallinity index (CrI) of CMC-based solid polymer electrolytes, a parameter that directly correlates with ionic transport properties. In the LiCH_3_COO system, the CrI values remain relatively high, with 43.73% for CMC/[BMIm]Cl/LiCH_3_COO (30 wt%) [100] and 45.48% for CMC/LiCH_3_COO (30 wt%) [98], indicating that the polymer matrix retains a more ordered structure, which restricts segmental motion and limits Li^+^ mobility. In contrast, the LiClO_4_-based systems exhibit significantly lower crystallinity, with CrI values of 7.05% for CMC (80%)/pectin (20%)/glycerol/LiClO_4_ (1 g) [101], 21.24% for CMC (50%)/MC (50%)/LiClO_4_ (10%) [46], 23.41% for CMC (50%)/PVA (50%)/glycerol (45 wt%)/LiClO_4_ (20 wt%) [80], and 28.54% for CMC (50%)/PVA (50%)/citric acid (15%)/LiClO_4_ (25 wt%) [17]. Meanwhile, systems with other salts show intermediate CrI values, namely 17.60% for CMC (0.2 g)/glycerol (0.08 g)/LiI (0.1 g), 19.26% for CMC/LiI (65 wt%) [70], and 29.47% for CMC (1.6 g)/PVA (0.4 g)/LiNO_3_ (20 wt%) [103].

These observations can be strongly supported by established theoretical frameworks in polymer electrolyte science. High crystallinity results in recrystalline chain mobility, thereby reducing ionic conductivity [104]. Conversely, reducing crystallinity through structural modifications or blending with amorphous polymers enhances segmental dynamics and ion transport [105,106]. The incorporation of plasticizers, such as glycerol, increases the fractional free volume in the polymer matrix, thereby enhancing chain flexibility and facilitating ion transport [107,108]. Ionic conduction in polymer electrolytes is coupled with the local motion of polymer chains; thus, reduced crystallinity enhances segmental dynamics and improves ionic conductivity [104,105].

The crystallinity of CMC-based solid polymer electrolytes plays a fundamental role in governing both ion transport and mechanical integrity. Although reducing crystallinity generally increases the amorphous fraction and facilitates segmental motion, excessive disruption of the ordered polymer structure may compromise dimensional stability and mechanical strength [109]. Conversely, highly crystalline regions improve structural rigidity but restrict polymer chain mobility, thereby limiting lithium-ion migration. Functional additives, therefore, act not only as fillers or plasticizers but also as regulators of the crystalline–amorphous balance [103]. The most effective electrolyte systems are those that maintain sufficient amorphous domains to promote ionic transport while preserving an interconnected polymer framework that supports mechanical and electrochemical stability.

### 6.3. Ionic Conductivity

**Figure 10:**
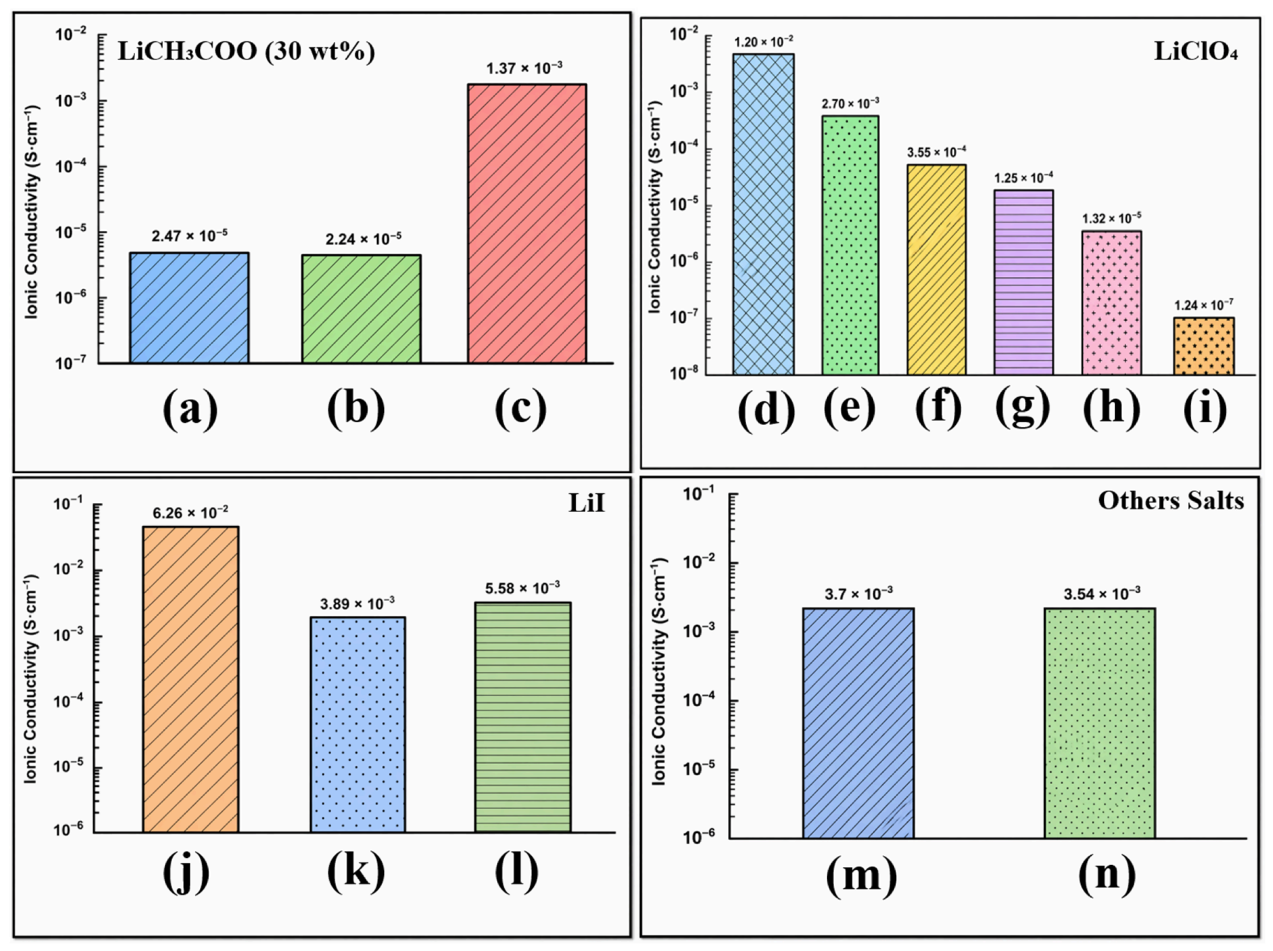
Effect of lithium salts and an additive on ionic conductivity: (a) CMC/LiCH_3_COO [98], (b) CMC/CMCh/LiCH_3_COO [8], (c) CMC/[BMIm]Cl/LiCH_3_COO [100], (d) CMC/MC/LiClO_4_ [46], (e) CMC/PVA/Gly/LiClO_4_ [80], (f) CMC/pectin/Gly/LiClO_4_ [101], (g) CMC/PVA/CA/LiClO_4_ [17], (h) CMC/LiClO_4_ [68], (i) CMC/CA/LiClO_4_ [18], (j) CMC/Gly/LiI [102], (k) CMC/CMKC/LiI [110], (l) CMC/LiI [70], (m) CMC/Gly/LiBF_4_ [15], and (n) CMC/PVA/LiNO_3_ [103].

The ionic conductivity of carboxymethyl cellulose (CMC)-based electrolytes shows a strong dependence on the salt type, polymer composition, and the presence of additives such as plasticizers, ionic liquids, and cross-linkers, as shown in Figure 10 and Table 3.

The comparative analysis presented in Figure 10 and Table 3 highlights the significant influence of lithium salt selection and additive incorporation on the ionic conductivity of carboxymethyl cellulose (CMC)-based polymer electrolytes. Across the compiled studies, ionic conductivity spans from 10^−7^ to 10^−2^ S cm^−1^, indicating that ion transport is highly sensitive to both the physicochemical properties of the salt and the structural modifications of the polymer matrix. In the LiCH_3_COO system, the incorporation of the ionic liquid [BMIm]Cl markedly enhances conductivity from ~2.47 × 10^−5^ to 1.37 × 10^−3^ S cm^−1^ [100], corresponding to an increase of nearly two orders of magnitude. This enhancement can be attributed to the dual role of ionic liquids as both plasticizing agents and ion-dissociation promoters, which reduce ion pairing and facilitate Li^+^ migration via segmental motion-assisted hopping mechanisms [111,112,113]. The interaction between the imidazolium cation and the –COO^−^ groups of CMC further disrupts interchain hydrogen bonding, flexibility, and free volume [114].

In contrast, the LiClO_4_-based systems exhibit a strong dependence on plasticizer content and polymer blending. The highest conductivity (~1.20 × 10^−2^ S cm^−1^) is achieved in systems containing urea or glycerol, whereas the absence of such additives results in a drastic decrease to ~10^−7^ S cm^−1^. This behavior is closely associated with the reduction in crystallinity and the expansion of the amorphous phase, which serves as the primary pathway for ion transport [115,116]. The large and weakly coordinating ClO_4_^−^ anion facilitates greater ion dissociation, thereby increasing the number of charge carriers [117,118]. However, excessive polymer blending (e.g., with PVA or pectin) may introduce structural rigidity, limiting segmental mobility and reducing ionic conductivity [80,101].

Notably, LiI-based systems demonstrate the highest conductivity values, reaching up to 6.26 × 10^−2^ S cm^−1^ in the presence of glycerol [102]. This superior performance is attributed to the high polarizability of the I^−^ anion, which weakens electrostatic interactions with Li^+^ and promotes efficient salt dissociation [119,120]. The resulting increase in charge-carrier density, combined with enhanced polymer chain dynamics from plasticization, enables efficient ion transport via both vehicular and hopping mechanisms. Nevertheless, this performance is highly composition-dependent, as evidenced by the reduced conductivity (~10^−3^ S cm^−1^) in blended systems, suggesting that microstructural constraints can offset the intrinsic advantages of the salt.

For other lithium salts such as LiBF_4_ and LiNO_3_, the observed conductivities (~3.5–3.7 × 10^−3^ S cm^−1^) indicate a moderate ion transport capability governed by the balance between anion size and coordination strength. The bulky and weakly coordinating BF_4_^−^ anion supports higher ionic mobility [15], while the planar NO_3_^−^ anion exhibits resonance stabilization that may slightly limit its dissociation efficiency [103]. Therefore, the rational design of high-performance solid polymer electrolytes requires the synergistic optimization of salt type, plasticizer content, and polymer architecture to achieve conductivity values approaching practical applicability in solid-state electrochemical devices.

Ionic transport in CMC-based solid polymer electrolytes is governed by the interplay between the segmental hopping mechanism and the vehicle mechanism. In relatively rigid polymer matrices, lithium ions migrate predominantly via coordination and hopping between neighboring oxygen-containing functional groups, which are associated with local polymer segmental motion [121]. In contrast, the incorporation of plasticizers or ionic liquids enhances free volume and salt dissociation, enabling solvated ions to migrate through the vehicle mechanism [78]. The dominant transport pathway, therefore, depends strongly on polymer flexibility, ion dissociation, and electrolyte microstructure. Rational additive design should optimize these competing mechanisms by promoting sufficient polymer mobility to facilitate Li^+^ hopping while preventing excessive plasticization that could reduce mechanical strength and long-term stability.

### 6.4. Electrochemical Stability

**Figure 11:**
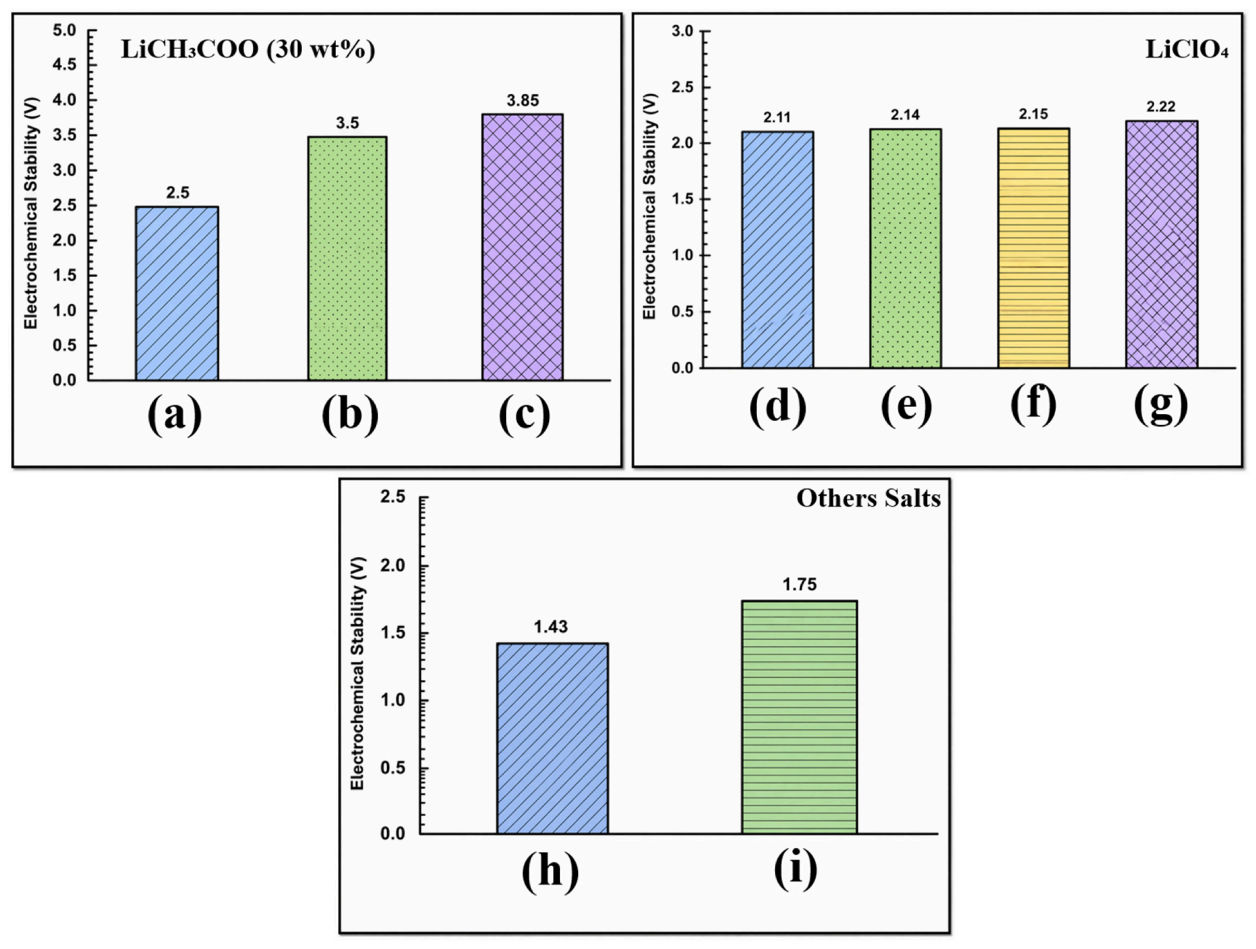
Effect of lithium salts and an additive on electrochemical stability: (a) CMC/CMCh/LiCH_3_COO [8], (b) CMC/LiCH_3_COO [98], (c) CMC/[BMIm]Cl/LiCH_3_COO [100], (d) CMC/PVA/CA/LiClO_4_ [17], (e) CMC/LiClO_4_ [68], (f) CMC/CA/LiClO_4_ [18], (g) CMC/PVA/Gly/LiClO_4_ [80], (h) CMC/PVA/LiNO_3_ [103], and (i) CMC/Gly/LiBF_4_ [15].

The electrochemical stability window (ESW), or anodic stability limit when only oxidative stability is evaluated, represents an important parameter for assessing the applicability of polymer electrolytes in electrochemical energy storage systems [122]. It defines the potential range over which the electrolyte remains stable against electrochemical decomposition under the experimental conditions employed. The electrochemical stability values reported for CMC-based polymer electrolytes, as summarized in Figure 11 and Table 4, demonstrate a strong dependence on both lithium salt chemistry and the presence of functional additives. Throughout this section, the reported values follow the terminology adopted in the original references.

In the LiCH_3_COO-based systems, the ESW increases significantly from 2.5 V in the CMC/CMCh blend to 3.85 V upon incorporation of the ionic liquid [BMIm]Cl. This improvement has been attributed to the incorporation of the ionic liquid, which has been reported to suppress electrolyte decomposition at higher potentials while improving interfacial compatibility with the electrodes [123,124]. Mechanistically, the imidazolium-based ionic liquid contributes to a more stable interphase by reducing localized charge accumulation and mitigating oxidative degradation, thereby extending the electrochemical window [92,125]. Additionally, the increased amorphous character induced by ionic liquid incorporation enhances the homogeneity of ion distribution, further stabilizing the system under applied potential.

In LiClO_4_-based systems, the electrochemical stability is relatively moderate but consistent, ranging from 2.11 to 2.22 V across various compositions. The slight increase in ESW with increasing salt concentration and plasticizer content (e.g., glycerol) suggests that ion–polymer interactions and matrix flexibility help stabilize the electrolyte against electrochemical decomposition [80]. The presence of citric acid as a cross-linking agent also contributes to improved structural integrity, thereby limiting segmental over-relaxation and reducing susceptibility to electrochemical breakdown [17]. However, compared to LiCH_3_COO systems in ionic liquids, the ESW values remain lower, likely due to the ClO_4_ anion’s relatively higher reactivity under oxidative conditions, despite its favorable dissociation characteristics [100].

For other lithium salts, such as LiNO_3_ and LiBF_4_, the ESW values are 1.43 V and 1.75 V, respectively, indicating comparatively lower electrochemical stability [15,103]. This behavior can be attributed to the intrinsic electrochemical properties of the anions. NO_3_^−^ is prone to reductive decomposition, while BF_4_^−^, although more stable, can undergo hydrolysis or side reactions under certain conditions, limiting its operational voltage range [126]. Furthermore, the absence of strong plasticizers or stabilizers in these systems reduces the polymer matrix’s ability to buffer electrochemical stress, resulting in narrower stability windows.

Improving the electrochemical stability window requires balancing ionic conductivity with oxidative and reductive stability. While additives such as ionic liquids and inorganic nanofillers can broaden the electrochemical stability window by stabilizing the polymer matrix and suppressing undesirable interfacial reactions, excessive additive loading may introduce structural heterogeneity or interfacial defects that adversely affect electrolyte performance [127,128]. Consequently, enhancing electrochemical stability should not be considered in isolation from other electrolyte properties, but rather as part of an integrated materials design strategy that simultaneously optimizes ionic transport, interfacial compatibility, and structural integrity.

### 6.5. Ion Transfer Number

**Figure 12:**
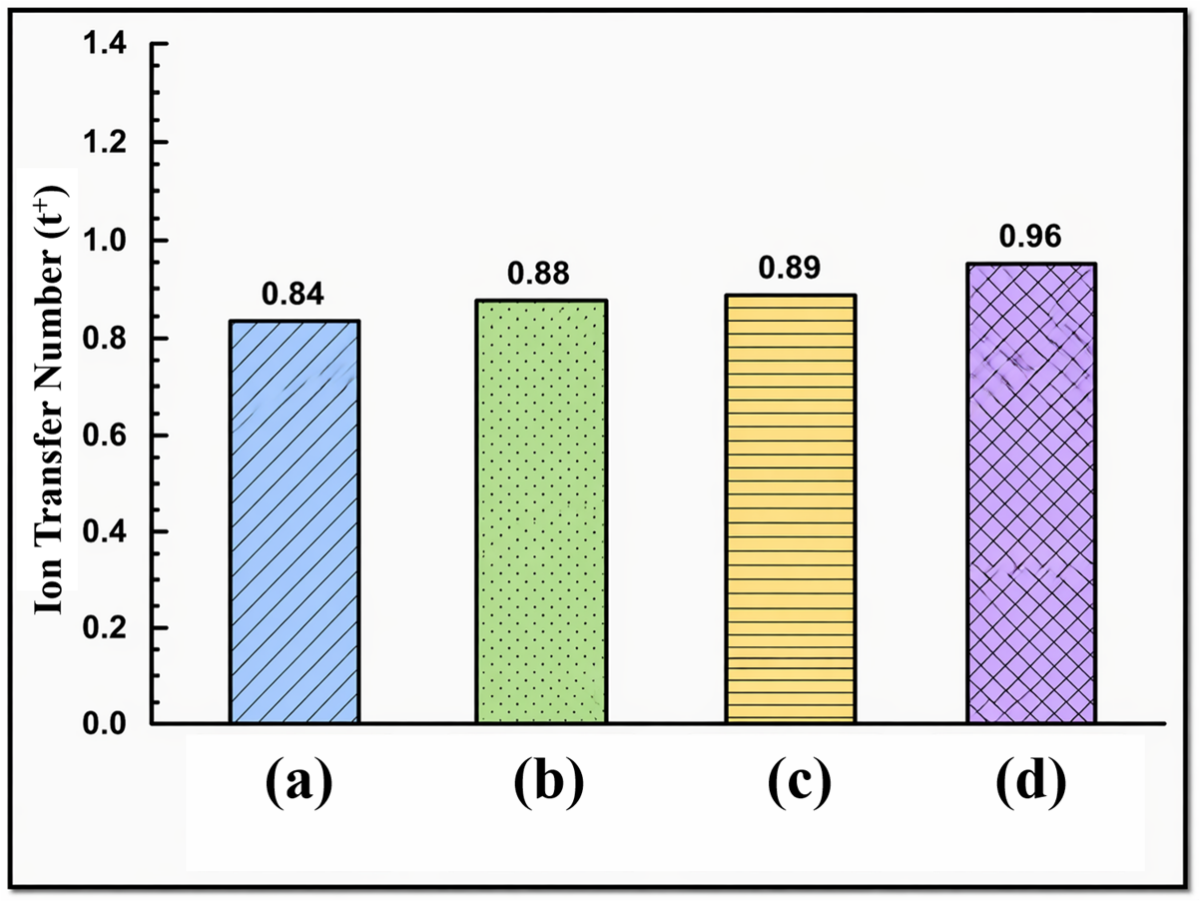
Effect of lithium salts and an additive on the ion transfer number: (a) CMC/CMCh/LiCH_3_COO [8], (b) CMC/PVA/CA/LiClO_4_ [17], (c) CMC/PVA/Gly/LiClO_4_ [80], and (d) CMC/[BMIm]Cl/LiCH_3_COO [100].

The ion transference number is a fundamental electrochemical parameter that quantifies the relative contribution of ionic species to the overall electrical conductivity of an electrolyte system. Specifically, it distinguishes the fraction of total current carried by ions from that carried by electrons, thereby providing critical insight into the dominant charge-transport mechanism within the material [129,130]. The ion transfer number (t^+^) is shown in Figure 12 and Table 5.

The ion transfer number (t^+^), as shown in Figure 12 and Table 5, provides critical insight into the dominant charge-transport mechanism in CMC-based polymer electrolytes. The reported t^+^ values range from 0.84 to 0.96, indicating that cationic transport (Li^+^) is the primary contributor to ionic conduction across all systems. Such high t^+^ values are particularly desirable for solid polymer electrolytes, as they minimize concentration polarization and enhance electrochemical performance in energy storage devices [131,132].

In the baseline system comprising CMC/CMCh with LiCH_3_COO (30 wt%), the t^+^ value of 0.84 suggests that, although Li^+^ transport dominates, a non-negligible contribution from anion mobility remains [8]. The presence of CMCh likely introduces additional hydrogen-bonding interactions, partially restricting Li^+^ mobility while allowing some degree of anion participation. Upon modification with polymer blending and cross-linking, as observed in the CMC/PVA system with citric acid and LiClO_4_, the t^+^ value increases to 0.88 [17]. This improvement can be attributed to the formation of a more structured polymer network through esterification reactions, which selectively suppresses anion mobility by trapping larger anions (ClO_4_^−^) within the polymer matrix, thereby enhancing the relative contribution of Li^+^ transport.

A further increase in t^+^ to 0.89 is observed in the presence of glycerol as a plasticizer [80]. Glycerol enhances segmental mobility of the polymer chains, facilitating Li^+^ hopping between coordination sites (–OH and –COO^−^ groups), while still maintaining partial immobilization of the anion due to its larger size and weaker interaction with the polymer backbone. This reflects a balanced mechanism in which polymer flexibility promotes cation transport without proportionally increasing anion diffusivity, a key requirement for achieving high-performance electrolytes [133,134].

The highest t^+^ value (0.96) is achieved in the system incorporating the ionic liquid [BMIm]Cl with LiCH_3_COO [100]. This near-unity cation transference number indicates a highly selective Li^+^ conduction pathway, which can be explained by strong interactions between the ionic liquid components and the polymer matrix. The bulky imidazolium cations and chloride anions are likely immobilized within the polymer network via electrostatic and hydrogen-bonding interactions, effectively suppressing their mobility. Concurrently, Li^+^ ions benefit from enhanced dissociation and increased availability of coordination sites, enabling efficient transport via a segmental motion-assisted hopping mechanism.

A high lithium-ion transference number is desirable because it minimizes concentration polarization and improves charge transport efficiency during battery operation. However, increasing the transference number alone does not necessarily guarantee superior electrolyte performance if accompanied by reduced ionic conductivity or limited polymer chain mobility [129]. Functional additives that selectively immobilize anions while maintaining efficient lithium-ion migration represent an effective strategy for enhancing electrolyte performance. Future material design should therefore focus on maximizing lithium-ion selectivity without compromising the overall ionic conductivity and mechanical stability of the polymer electrolyte.

### 6.6. Mechanical Properties

**Figure 13:**
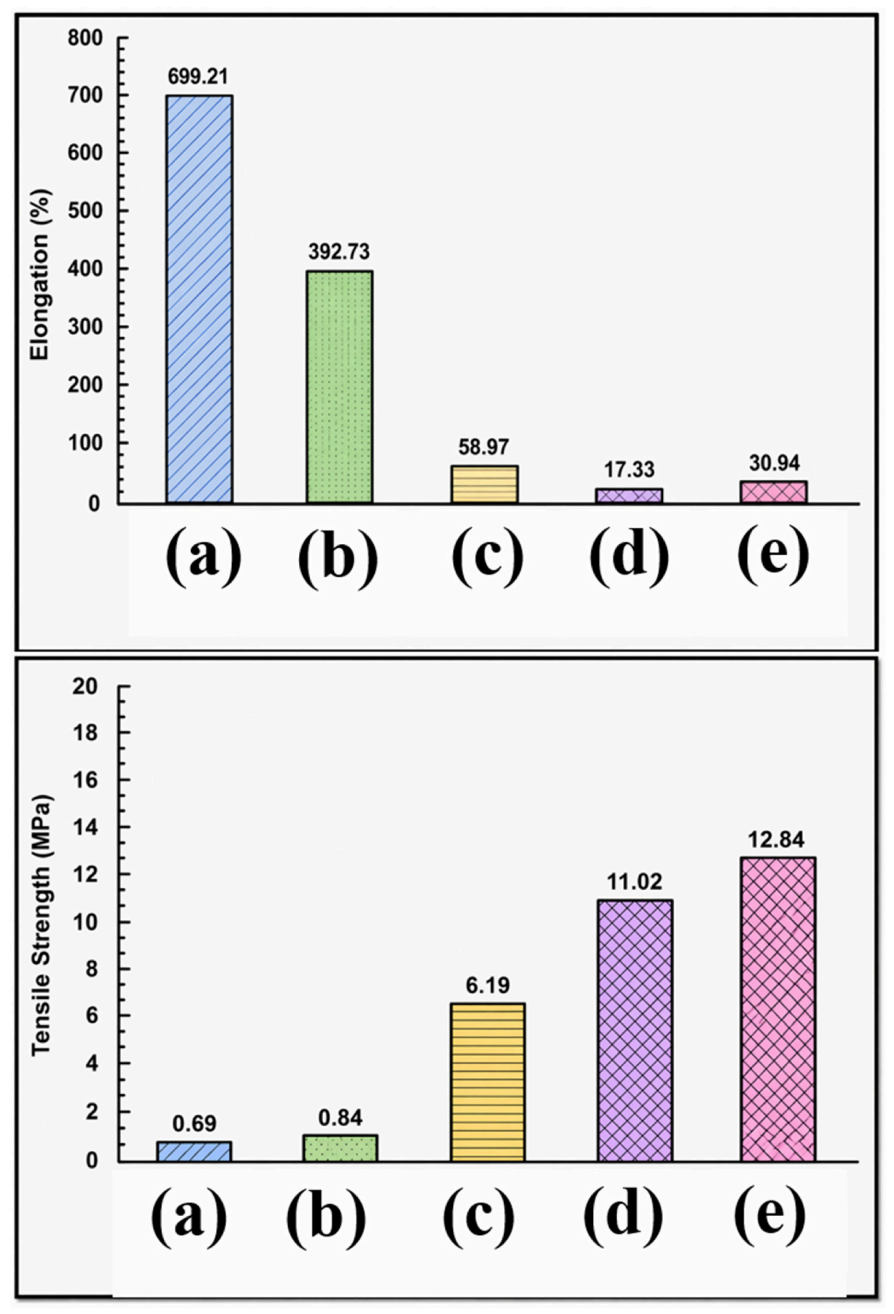
Effect of lithium salts and an additive on mechanical properties: (a) CMC/PVA/CA/LiClO_4_ [17], (b) CMC/PVA/Gly/LiClO_4_ [80], (c) CMC/CMCh/LiCH_3_COO [8], (d) CMC/MC/LiClO_4_ [46], and (e) CMC/LiCH_3_COO [98].

Mechanical properties are a critical determinant of the practical applicability of solid polymer electrolytes (SPEs), as they govern structural integrity, flexibility, and resistance to deformation under operational conditions. The effect of lithium salts and additives on mechanical properties is shown in Figure 13 and Table 6.

The mechanical properties of CMC-based polymer electrolytes, as illustrated in Figure 13 and Table 6, reveal a pronounced trade-off between flexibility (elongation at break) and mechanical strength (tensile strength), governed by the interplay between polymer blending, plasticization, and cross-linking. The elongation values range from 17.33% to 699.21%, while tensile strength ranges from 0.69 to 12.84 MPa, indicating that compositional modifications strongly influence the system’s structural integrity and deformability.

In the CMC/PVA system containing citric acid and LiClO_4_, an exceptionally high elongation of 699.21% is observed, accompanied by a relatively low tensile strength of 0.69 MPa [17]. This behavior is characteristic of highly plasticized and loosely cross-linked polymer networks, where the presence of glycerol and ester-type cross-links enhances chain mobility and free volume. The increased flexibility arises from reduced intermolecular forces and disruption of crystalline domains, allowing polymer chains to undergo extensive deformation before failure [135,136]. However, this enhanced segmental motion compromises mechanical strength, as the network lacks sufficient rigidity to resist applied stress.

A similar trend is observed in the CMC/PVA system with glycerol, where elongation remains high (392.73%), but tensile strength slightly increases to 0.84 MPa [80]. Glycerol present in the system acts as a plasticizer, reducing intermolecular interactions and promoting ductility while maintaining a moderate level of structural cohesion. These findings suggest that plasticizer-rich systems are advantageous for achieving flexible electrolytes but may require reinforcement for mechanical stability in practical applications [137,138].

In contrast, systems based on CMC/CMCh and CMC/MC exhibit significantly reduced elongation (58.97% and 17.33%, respectively) but markedly improved tensile strength (6.19 and 11.02 MPa) [8,46]. This indicates a transition toward more rigid, compact polymer networks, in which strong intermolecular interactions, such as hydrogen bonding and chain entanglement, restrict polymer mobility. The incorporation of MC (methyl cellulose) enhances structural packing and stiffness, thereby increasing resistance to mechanical deformation. Additionally, the presence of LiClO_4_ may contribute to physical cross-linking through ion–dipole interactions, further reinforcing the system.

The highest tensile strength (12.84 MPa) is achieved in the CMC system with LiCH_3_COO, despite a moderate elongation of 30.94% [98]. This suggests that the system exhibits an optimal balance between rigidity and flexibility, likely due to strong coordination between Li^+^ ions and the carboxylate groups (–COO^−^) of CMC. These interactions act as transient cross-linking points, enhancing mechanical strength without completely suppressing chain mobility. Such behavior is consistent with the concept of ionically cross-linked polymer networks, where reversible interactions contribute to both strength and toughness [45,64].

From a materials engineering perspective, high-performance CMC-based polymer electrolytes require an optimal balance between flexibility and rigidity, rather than maximizing either property in isolation [17]. Increasing crosslinking density generally enhances tensile strength, dimensional stability, and thermal resistance by restricting polymer chain mobility [139]. However, excessive crosslinking suppresses segmental motion, thereby reducing ionic conductivity. Conversely, plasticizers and flexible polymer blends enhance ductility and facilitate ion transport by increasing free volume, although excessive plasticization often reduces mechanical strength and dimensional stability. Consequently, the design of CMC-based polymer electrolytes should carefully optimize crosslinking density, along with the type and concentration of functional additives, to achieve a synergistic balance between electrochemical performance and mechanical robustness.

### 6.7. Degradation Temperature

**Figure 14:**
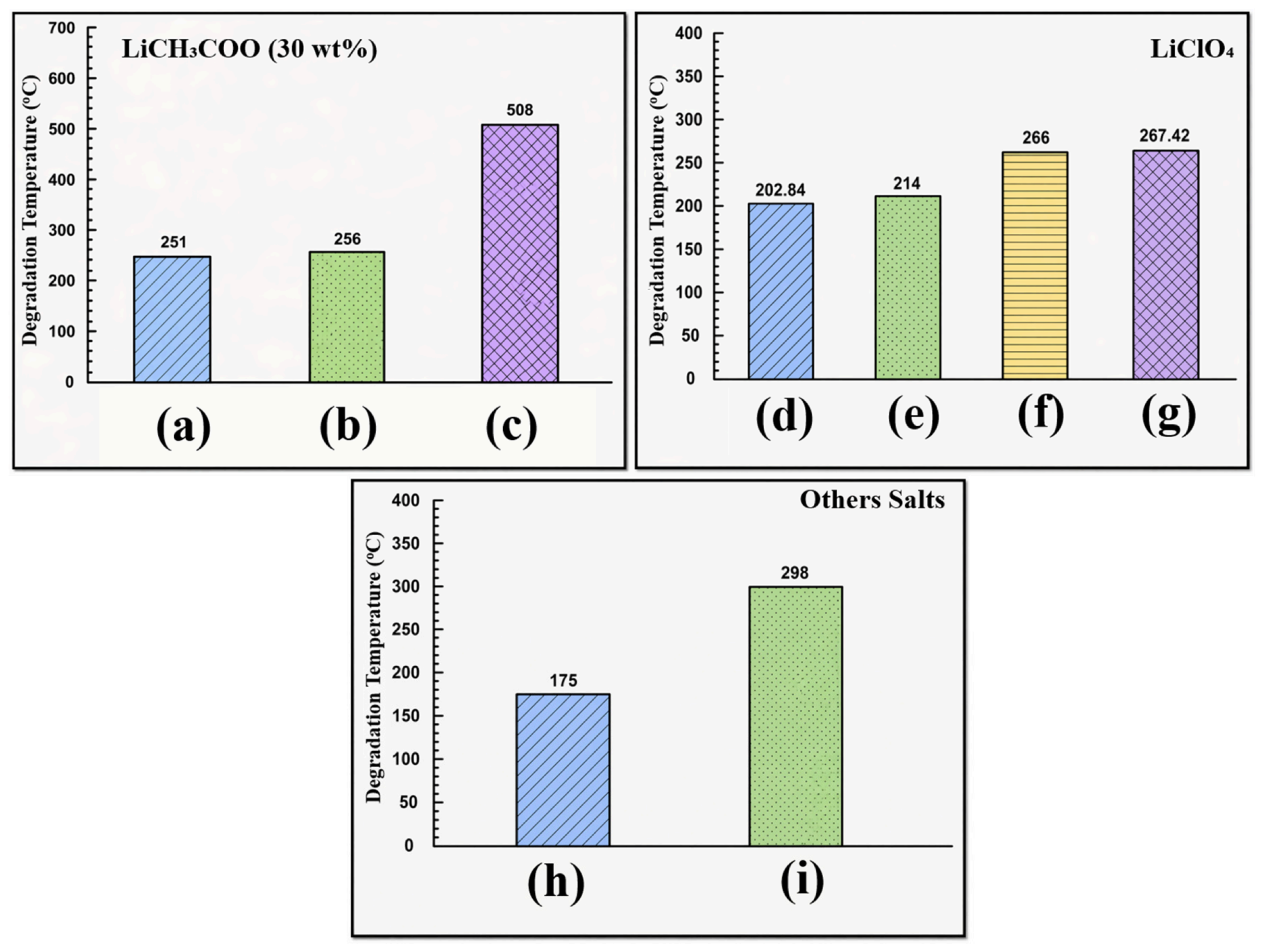
Effect of lithium salts and an additive on degradation temperature: (a) CMC/LiCH_3_COO [98], (b) CMC/CMCh/LiCH_3_COO [8], (c) CMC/[BMIm]Cl/LiCH_3_COO [100], (d) CMC/MC/LiClO_4_ [46], (e) CMC/pectin/Gly/LiClO_4_ [101], (f) CMC/PVA/Gly/LiClO_4_ [80], (g) CMC/PVA/CA/LiClO_4_ [17], (h) CMC/LiI [70], and (i) CMC/Gly/LiBF_4_ [15].

Degradation temperature is a crucial parameter for evaluating the safety and operational reliability of solid polymer electrolytes (SPEs), as it determines the material’s ability to maintain its structural and electrochemical integrity at elevated temperatures. The effects of lithium salts and additives on the degradation temperature are shown in Figure 14.

The degradation temperature of CMC-based polymer electrolytes, as shown in Figure 14 and Table 7, strongly depends on the type of lithium salt and the incorporation of functional additives, with degradation temperatures ranging from approximately 175 °C to 508 °C. This wide variation reflects the critical roles of polymer–salt interactions, cross-linking density, and structural modifications in determining the system’s thermal resistance.

In the LiCH_3_COO-based systems, degradation temperature increases from 251 °C in the pristine CMC matrix [98] to 256 °C in the CMC/CMCh blend [8], and further increases to a remarkably high value of 508 °C upon incorporation of the ionic liquid [BMIm]Cl [100]. The dramatic enhancement observed with ionic liquid addition indicates the formation of a more thermally robust network, likely due to strong electrostatic interactions and improved dispersion of ionic species within the polymer matrix. The ionic liquid not only promotes better ion dissociation but also stabilizes the polymer chains by reducing segmental mobility at elevated temperatures, thereby delaying thermal degradation [140]. Additionally, the presence of bulky imidazolium ions may hinder chain scission, thereby enhancing degradation temperature [141,142].

For LiClO_4_-based systems, the degradation temperature ranges from 202.84 °C to 267.42 °C, depending on the composition. Systems containing glycerol and polymer blends, such as PVA and pectin, exhibit greater stability than simpler matrices. This is attributed to the formation of hydrogen-bonding networks and partial cross-linking, which reinforce the polymer’s structural integrity [45,143,144]. The highest value (267.42 °C) observed in the CMC/PVA system with citric acid suggests that chemical cross-linking via esterification significantly enhances degradation temperature by restricting polymer chain mobility and increasing resistance to decomposition [17]. However, the presence of plasticizers such as glycerol may introduce competing effects, in which increased flexibility can lower the onset temperature of thermal degradation despite improving other functional properties.

In systems containing other lithium salts, such as LiI and LiBF_4_, the thermal stabilities are moderate at 175 °C and 298 °C, respectively [15,103]. The relatively lower stability of the LiI system may be associated with the iodide anion’s higher reactivity and lower degradation temperature, which can facilitate earlier degradation pathways. In contrast, LiBF_4_ exhibits a higher degradation temperature due to its more stable anion structure and stronger interactions with the polymer matrix, particularly in the presence of glycerol, which enhances structural homogeneity and reduces localized thermal stress.

Thermal stability is closely associated with the strength of intermolecular interactions within the polymer electrolyte rather than the intrinsic properties of individual components alone. Crosslinking, hydrogen bonding, and the incorporation of thermally stable inorganic fillers generally improve resistance to thermal degradation by reinforcing the polymer network [38]. Nevertheless, excessive incorporation of additives may alter the polymer microstructure and reduce the flexibility required for efficient ion transport. Therefore, future development of CMC-based solid polymer electrolytes should emphasize the use of multifunctional additives that simultaneously enhance thermal stability, mechanical properties, and ionic conductivity without introducing adverse trade-offs.

## 7. Summary and Outlook

Despite substantial progress in the development of carboxymethyl cellulose (CMC)-based solid polymer electrolytes (SPEs), several important challenges must be addressed before these materials can be translated into practical energy storage applications. The overall performance of CMC-based SPEs is governed by a complex interplay among polymer structure, lithium salt chemistry, and functional additives, making it difficult to simultaneously achieve high ionic conductivity, sufficient mechanical strength, wide electrochemical stability, and long-term durability. Although numerous modification strategies, including polymer blending, cross-linking, plasticization, nanofiller incorporation, and ionic liquid addition, have demonstrated significant performance improvements, the synergistic effects among these approaches have yet to be optimized systematically. Furthermore, the molecular mechanisms governing ion dissociation, Li^+^ transport, and polymer–ion interactions are not fully understood, and issues related to long-term stability, electrode–electrolyte interfacial compatibility, and scalable manufacturing continue to limit practical implementation.

The comparison presented in Table 8 demonstrates that no single CMC-based solid polymer electrolyte simultaneously achieves the highest ionic conductivity, mechanical strength, electrochemical stability, and thermal stability. Plasticizers and ionic liquids generally enhance ionic conductivity by increasing polymer chain mobility and promoting lithium salt dissociation, whereas inorganic nanofillers and crosslinking agents primarily improve mechanical integrity and thermal stability. Consequently, future electrolyte design should emphasize multifunctional material systems that simultaneously optimize ion transport, structural robustness, and electrochemical durability, rather than maximizing a single performance parameter.

Compared with other naturally derived polymer electrolytes, CMC offers a unique combination of abundant hydroxyl and carboxymethyl functional groups, excellent film-forming capability, environmental sustainability, and high compatibility with various lithium salts and functional additives (Table 9). These characteristics enable flexible molecular design through plasticization, crosslinking, polymer blending, and nanofiller incorporation [10]. Nevertheless, like other biopolymer electrolytes, CMC still faces challenges, including relatively low room-temperature ionic conductivity, moisture sensitivity, and an inherent trade-off between ionic conductivity and mechanical robustness [9]. Therefore, future developments should focus on integrating the advantages of different biopolymer matrices while exploiting the tunable chemistry of CMC to achieve balanced electrochemical and mechanical performance.

Future research should therefore adopt a more integrated approach combining rational material design with advanced characterization and electrochemical evaluation. Optimizing the composition of polymer matrices, lithium salts, and multifunctional additives is expected to provide a better balance between ionic conductivity, mechanical robustness, and electrochemical stability. Additionally, greater emphasis should also be placed on understanding structure–property relationships through multiscale experimental and computational approaches, enabling the rational design of next-generation polymer electrolytes. Future studies should extend beyond laboratory-scale material development by evaluating long-term cycling performance, interfacial stability with electrode materials, and the overall environmental sustainability of the complete electrolyte system. Although CMC is a renewable and biodegradable polymer, the sustainability of CMC-based solid polymer electrolytes also depends on the environmental impacts of lithium salts, ionic liquids, inorganic nanofillers, solvents, and processing methods, as well as considerations related to material safety, recyclability, and end-of-life management. Therefore, future research should incorporate life-cycle-oriented design strategies that balance electrochemical performance with environmental responsibility. Addressing these challenges will accelerate the development of high-performance, sustainable, and commercially viable CMC-based solid polymer electrolytes for advanced energy storage systems.

## Figures and Tables

**Table 1:** Comparison of previous review articles about CMC-based polymer electrolytes and the present review.

Author (Year)	Scope	Main Focus	Limitations	Ref.
Arif et al. (2025)	CMC membranes	Preparation and applications	This review is limited to CMC-based SPEs with various ionic salts and does not comprehensively address the influence of lithium salts and other additives in lithium-based SPEs.	[10]
Tyagi et al. (2023)	CMC/PVA	Materials and applications	Only discuss the broad application, not structure or properties	[11]
Akhlaq et al. (2023)	CMC materials	Materials and application	Focuses mainly on electrochemical properties, with limited discussion of mechanical and thermal properties.	[12]
This review	CMC SPE	Systematic analysis	Structure–property–performance relationships	This work

**Table 2:** Inclusion and Exclusion Criteria Used for Study Selection.

Criteria	Inclusion Criteria	Exclusion Criteria
Database	Scopus-indexed publications	Other databases
Publication type	Original peer-reviewed research articles	Reviews, conference papers, editorials, book chapters, notes, short communications
Language	English	Non-English publications
Polymer system	CMC as the primary polymer matrix for solid polymer electrolytes (SPEs)	Gel polymer electrolytes, hydrogels, separators, non-CMC systems, or CMC used only as an additive
Electrolyte system	Lithium salt-based SPEs (e.g., LiClO_4_, LiBF_4_, LiTFSI, LiPF_6_, LiCF_3_SO_3_, LiCH_3_COO, LiI, LiNO_3_)	Sodium-, potassium-, magnesium-, zinc-, ammonium-, or proton-based electrolytes
Application	Lithium-ion batteries or lithium-based energy storage	Supercapacitors, fuel cells, sensors, biomedical, or other non-battery applications
Characterization	Reports at least one electrochemical or physicochemical property (e.g., ionic conductivity, electrochemical stability, crystallinity, thermal or mechanical properties)	Insufficient experimental characterization or no quantitative electrochemical data
Accessibility	Full-text available	Abstract only or inaccessible full text
Quality	Complete methodology and reproducible quantitative results	Incomplete methods or ambiguous findings
Final selection	Meets all inclusion criteria after full-text assessment	Fails one or more inclusion criteria

**Table 3:** Comparison of Electrochemical Impedance Spectroscopy (EIS) Conditions and Ionic Conductivity of CMC-Based Polymer Electrolytes.

Code	Polymer Electrolyte Composition	EIS Frequency Range	Temperature (K)	Electrode Configuration	Cell Type	Ionic Conductivity (S cm^−1^)	Ref.
(a)	CMC/LiCH_3_COO	42 Hz–1 MHz	298	Cu/SPE/Cu	Blocking	2.47 × 10^−5^	[98]
(b)	CMC/CMCh/LiCH_3_COO	42 Hz–1 MHz	298	Cu/SPE/Cu	Blocking	2.24 × 10^−5^	[8]
(c)	CMC/[BMIm]Cl/LiCH_3_COO	42 Hz–1 MHz	298	Cu/SPE/Cu	Blocking	1.37 × 10^−3^	[100]
(d)	CMC/MC/LiClO_4_	42 Hz–1 MHz	298	Cu/SPE/Cu	Blocking	1.20 × 10^−2^	[46]
(e)	CMC/PVA/Gly/LiClO_4_	42 Hz–1 MHz	298	Cu/SPE/Cu	Blocking	2.70 × 10^−3^	[80]
(f)	CMC/Pectin/Gly/LiClO_4_	10 Hz–500 kHz	303–353	Stainless steel	Swagelok cell	3.55 × 10^−4^	[101]
(g)	CMC/PVA/CA/LiClO_4_	42 Hz–1 MHz	298	Cu/SPE/Cu	Blocking	1.25 × 10^−4^	[17]
(h)	CMC/LiClO_4_	0.1 Hz–50 MHz	298	Li/SPE/Li	Symmetrical Li cell	1.32 × 10^−5^	[68]
(i)	CMC/CA/LiClO_4_	0.1 Hz–50 MHz	298	Li/SPE/Li	Symmetrical Li cell	1.24 × 10^−7^	[18]
(j)	CMC/Gly/LiI	50 Hz–5 MHz	303–353	NA	NA	6.26 × 10^−2^	[102]
(k)	CMC/CMKC/LiI	10 Hz–4 MHz	300–333	Stainless steel	Blocking	3.89 × 10^−3^	[110]
(l)	CMC/LiI	50 Hz–5 MHz	303–353	NA	NA	5.58 × 10^−3^	[70]
(m)	CMC/Gly/LiBF_4_	10 Hz–1 MHz	298	NA	NA	3.7 × 10^−3^	[15]
(n)	CMC/PVA/LiNO_3_	50 Hz–1 MHz	303–343	SS/SPE/SS	Blocking	3.54 × 10^−3^	[103]

**Table 4:** Linear Sweep Voltammetry (LSV) Conditions and Electrochemical Stability of CMC-Based Solid Polymer Electrolytes.

Code	Polymer Electrolyte Composition	Technique	Temperature (°C)	Scan Rate	Electrode Configuration	Electrochemical Stability Window (V)	Ref.
(a)	CMC/CMCh/LiCH_3_COO	LSV	25	1 mV s^−1^	SS/SPE/Li	2.5	[8]
(b)	CMC/LiCH_3_COO	LSV	25	1 mV s^−1^	SS/SPE/Li	3.5	[98]
(c)	CMC/[BMIm]Cl/LiCH_3_COO	LSV	25	500 mA (0.5 C)	SS/SPE/Li	3.85	[100]
(d)	CMC/PVA/CA/LiClO_4_	LSV	25	500 mA (0.5 C)	SS/SPE/Li	2.11	[17]
(e)	CMC/LiClO_4_	LSV	25	5 mV s^−1^	SS/SPE/Li	2.14	[68]
(f)	CMC/CA/LiClO_4_	LSV	25	5 mV s^−1^	SS/SPE/Li	2.15	[18]
(g)	CMC/PVA/Gly/LiClO_4_	LSV	25	500 mA (0.5 C)	SS/SPE/Li	2.22	[80]
(h)	CMC/PVA/LiNO_3_	LSV	30–70	10 mV s^−1^	SS/SPE/SS	1.43	[103]
(i)	CMC/Gly/LiBF_4_	LSV	25	50 mV s^−1^	SS/SPE/Li	1.75	[15]

**Table 5:** Experimental Conditions and Lithium-Ion Transference Number Measurements of CMC-Based Polymer Electrolytes.

Code	Polymer Electrolyte Composition	Measurement Technique	Applied DC Voltage (mV)	Polarization Time (s)	Temperature (°C)	Electrode Configuration	Lithium-Ion Transference Number (tLi^+^)	Ref.
(a)	CMC/CMCh/LiCH_3_COO	DC Polarization	10	1000	25	Li/SPE/Li	0.84	[8]
(b)	CMC/PVA/CA/LiClO_4_	DC Polarization	10	1000	25	Li/SPE/Li	0.88	[17]
(c)	CMC/PVA/Gly/LiClO_4_	DC Polarization	10	1000	25	Li/SPE/Li	0.89	[80]
(d)	CMC/[BMIm]Cl/LiCH_3_COO	DC Polarization	10	1000	25	Li/SPE/Li	0.96	[100]

**Table 6:** Experimental Conditions for Tensile Testing of CMC-Based Polymer Electrolytes.

Code	Polymer Electrolyte Composition	Testing Standard/Instrument	Load Cell (N)	Crosshead Speed (mm min^−1^)	Specimen Dimensions (mm^2^)	Elongation (%)	Strength (MPa)	Ref.
(a)	CMC/PVA/CA/LiClO_4_	UTM	5000	10	50 × 10	699.21	0.69	[17]
(b)	CMC/PVA/Gly/LiClO_4_	UTM	5000	10	50 × 10	392.73	0.84	[80]
(c)	CMC/CMCh/LiCH_3_COO	UTM	5000	10	50 × 10	58.97	6.19	[8]
(d)	CMC/MC/LiClO_4_	UTM	5000	10	50 × 10	17.33	11.02	[46]
(e)	CMC/LiCH_3_COO	UTM	5000	10	50 × 10	30.94	12.84	[98]

**Table 7:** Thermal Characterization Conditions of CMC-Based Polymer Electrolytes.

Code	Polymer Electrolyte Composition	Methods	Heating Rate (°C min^−1^)	Temperature Range (°C)	Atmosphere	Degradation Temperature	Ref.
(a)	CMC/LiCH_3_COO	TGA	10	29–600	N_2_	251	[98]
(b)	CMC/CMCh/LiCH_3_COO	TGA	10	29–600	N_2_	256	[8]
(c)	CMC/[BMIm]Cl/LiCH_3_COO	TGA	10	29–600	N_2_	508	[100]
(d)	CMC/MC/LiClO_4_	TGA	10	29–600	N_2_	202.84	[46]
(e)	CMC/Pectin/Gly/LiClO_4_	TGA	NA	25–800	N_2_	214	[101]
(f)	CMC/PVA/Gly/LiClO_4_	TGA	10	25–600	N_2_	266	[80]
(g)	CMC/PVA/CA/LiClO_4_	TGA	10	25–600	N_2_	267.42	[17]

**Table 8:** Comparison of representative CMC-based solid polymer electrolyte systems reported in the literature.

Electrolyte System	Functional Additive	Max Ionic Conductivity (S·cm^−1^)	Thermal Stability (°C)	Tensile Strength (MPa)	Main Advantage	Ref.
CMC/Gly/LiBF_4_	Plasticizer	3.7 × 10^−3^	298	–	Plasticized SPE with enhanced ion transport	[15]
CMC/LiCH_3_COO	–	2.47 × 10^−5^	251	12.84	Good mechanical strength	[98]
CMC/CMCh/LiCH_3_COO	Blend Polymer	2.24 × 10^−5^	256	6.19	Polymer blending improves flexibility	[8]
CMC/[BMIm]Cl/LiCH_3_COO	Ionic liquid	1.37 × 10^−3^	508	–	Excellent thermal stability and conductivity	[100]
CMC/Pectin/Gly/LiClO_4_	Plasticizer	3.55 × 10^−4^	214	–	Biopolymer blend electrolyte	[101]
CMC/LiClO_4_	–	1.32 × 10^−5^	–	–	Simple CMC-based SPE	[68]
CMC/MC/LiClO_4_	Blend Polymer	1.20 × 10^−2^	202.84	11.02	Highest ionic conductivity	[46]
CMC/PVA/Gly/LiClO_4_	Plasticizer	2.70 × 10^−3^	266	0.84	Flexible polymer blend	[80]
CMC/CA/LiClO_4_	Crosslinker	1.24 × 10^−7^	–	–	Crosslinked electrolyte	[18]
CMC/PVA/CA/LiClO_4_	Crosslinker	1.25 × 10^−4^	267.42	0.69	Improved mechanical integrity	[17]
CMC/LiI	Plasticizer	6.26 × 10^−2^	–	–	Highest ionic conductivity among reviewed studies	[70]
CMC/CMKC/LiI	Blend Polymer	3.89 × 10^−3^	–	–	Carrageenan blend enhances conductivity	[110]
CMC/LiI	–	5.58 × 10^−3^	175	–	High LiI loading improves ion transport	[70]
CMC/PVA/LiNO_3_	Blend Polymer	3.54 × 10^−3^	–	–	Suitable for electrochemical energy storage	[103]

**Table 9:** Comparison of representative biopolymer-based solid polymer electrolytes for lithium batteries.

Polymer	Lithium Salt	Ionic Conductivity	Representative Battery/Device	Major Advantage (Conductivity)	Major Limitation (Conductivity)	Ref.
CMC	LiClO_4_ (10%)	1.32 × 10^−5^ S cm^−1^ (298 K)	Li-ion battery SPE	Moderate ionic conductivity at room temperature	Insufficient for high-performance batteries without modification	[18]
Pectin	LiClO_4_ (40%)	5.15 × 10^−5^ (303 K); 2.28 × 10^−4^ S cm^−1^ (353 K)	Li-ion battery SPE	Conductivity increases significantly with temperature	Limited room-temperature conductivity	[128]
Starch	LiClO_4_ (40%)	1.55 × 10^−6^ (298 K); 1.28 × 10^−4^ S cm^−1^ (353 K)	Li-ion battery SPE	High conductivity at elevated temperature	Very low conductivity at room temperature	[145]
Gelatin	LiClO_4_ (25%)	(3.58 ± 0.58) × 10^−6^ S cm^−1^ (298 K)	Li-ion battery SPE	Stable ion transport in a biopolymer matrix	Low room-temperature conductivity	[146]
Cellulose acetate	LiClO_4_ (16%)	4.9 × 10^−3^ S cm^−1^ (298 K)	Li-ion battery SPE	Highest room-temperature conductivity	Still lower than liquid electrolytes	[147]

## Data Availability

The original contributions presented in this study are included in the article. Further inquiries can be directed to the corresponding author.
